# Molecular Mobility and Gas Transport Properties of Mixed Matrix Membranes Based on PIM-1 and a Phosphinine Containing Covalent Organic Framework

**DOI:** 10.1021/acs.macromol.3c02419

**Published:** 2024-02-09

**Authors:** Farnaz Emamverdi, Jieyang Huang, Negar Mosane Razavi, Michael J. Bojdys, Andrew B. Foster, Peter M. Budd, Martin Böhning, Andreas Schönhals

**Affiliations:** † Bundesanstalt für Materialforschung und -prüfung (BAM), Unter den Eichen 87, Berlin 12205, Germany; ‡ Department of Chemistry, Humboldt University, Brook-Taylor Straße 2, Berlin 12489, Germany; § School of Chemistry, University of Manchester, Manchester M 13 9PL, United Kingdom

## Abstract

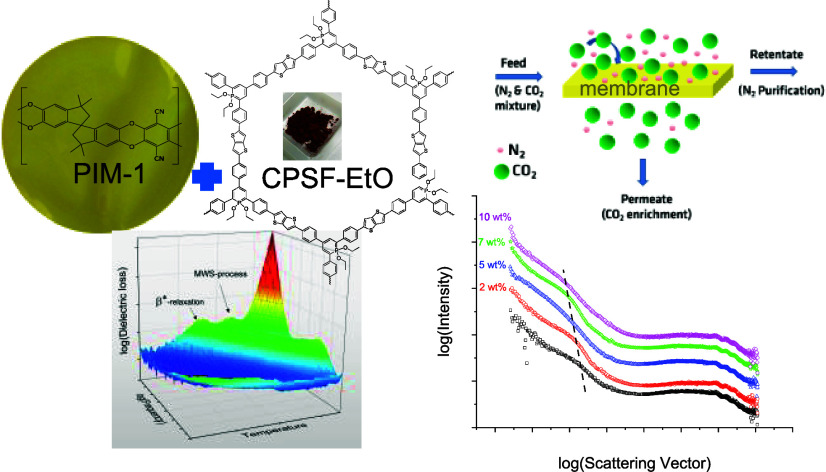

Polymers with intrinsic microporosity (PIMs) are gaining attention as gas separation membranes. Nevertheless, they face limitations due to their pronounced physical aging. In this study, a covalent organic framework containing λ^5^-phosphinine moieties, CPSF-EtO, was incorporated as a nanofiller (concentration range 0–10 wt %) into a PIM-1 matrix forming dense films with a thickness of ca. 100 μm. The aim of the investigation was to investigate possible enhancements of gas transport properties and mitigating effects on physical aging. The incorporation of the nanofiller occurred on an nanoaggregate level with domains up to 100 nm, as observed by T-SEM and confirmed by X-ray scattering. Moreover, the X-ray data show that the structure of the microporous network of the PIM-1 matrix is changed by the nanofiller. As molecular mobility is fundamental for gas transport as well as for physical aging, the study includes dielectric investigations of pure PIM-1 and PIM-1/CPSF-EtO mixed matrix membranes to establish a correlation between the molecular mobility and the gas transport properties. Using the time-lag method, the gas permeability and the permselectivity were determined for N_2_, O_2_, CH_4_, and CO_2_ for samples with variation in filler content. A significant increase in the permeability of CH_4_ and CO_2_ (50% increase compared to pure PIM-1) was observed for a concentration of 5 wt % of the nanofiller. Furthermore, the most pronounced change in the permselectivity was found for the gas pair CO_2_/N_2_ at a filler concentration of 7 wt %.

## Introduction

Global emissions of greenhouse gases, mainly arising from excessive combustion of fossil fuels, have increased significantly over the last decades and by causing climate change have become one of the most challenging environmental issues. Carbon dioxide (CO_2_) is currently one of the largest contributors to the greenhouse effect. Membrane technology is considered a separation technology with lower energy consumption compared to other conventional techniques providing a way for a sustainable industrial growth today and in the future, but also as a powerful tool for CO_2_ capture and storage (CCS) applications.^1,2^ Polymeric gas separation membranes have been already commercialized to replace conventional, energy-intensive processes such as pressure swing absorption or cryogenic distillation.^3,4^ In contrast to conventional separation processes like cryogenic distillation, membrane-based gas separation does not require a phase change.^2^

Membrane-based separations can be used in a wide range of process environments, such as in large-scale plants for purification of natural gas. Moreover, membrane-based separations are also suited for remote locations, where small-scale and simple units are preferred, such as in off-shore gas processing platforms or in local biogas plants.^1,5^ Additionally, gas separation processes employing membranes result in low operational costs.^6^

The basis of a separation membrane is the active separation layer.^7^ Many polymers, both in the rubbery and glassy state, have been studied for applications as an active separation layer. Among them are glassy polymers, like high free volume polyacetylenes and fluorinated materials.^8^ Moreover, high-performance glassy polymers, such as polyamides, polyimides, poly(amide-imides), polycarbonates, polysulfones, and microporous polynorbornenes as well as polymers of intrinsic microporosity (PIMs) have been investigated for that purpose (see for instance refs (9−17)).

The permeability and the permselectivity characterize the separation efficiency of a membrane.^7^ The permeability (*P*) of a gas through a dense polymer is based on the solution-diffusion mechanism.^18^ According to this mechanism, the permeation of small penetrant molecules through a membrane is controlled by two major parameters, the diffusivity (*D*) and the solubility (*S*).^11^ The permeability is then given by
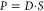
1

The ideal permselectivity (selectivity) α_A/B_ for a gas pair of two gas molecules A/B is defined by the ratio of their gas permeabilities *P*_*A*_ and *P*_*B*_
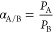
2

For polymeric membrane materials, Robeson recognized a trade-off relationship between the selectivity for a gas pair and the permeability.^19^ The higher the permeability, the lower is the selectivity. This trade-off relationship results in an empirical upper bound depicted in the so-called Robeson plot where log α_*A*/*B*_ is plotted versus log *P*_*A*_ of the more permeable gas. With time the upper bound has been revised to higher values of permeability and selectivity due to the development of new and improved polymer materials.^20−22^

Following the theory of Freeman describing a physicochemical basis of this phenomenological trade-off behavior, increasing the rigidity of polymer chains will enhance the membrane selectivity.^23^ This suggestion has been incorporated into the design criteria for the further development of polymers of intrinsic microporosity. These PIMs, glassy polymers with extremely high fractional free volume (FFV), introduced by Budd and McKeown in 2004^24^ represent an important class of functional high-performance polymers and are recognized as promising materials for membrane-based gas separation membranes. This is due to their chemical and thermal stability, with their high free volume forming an interconnected microporous structure leading to high permeability values together with reasonable permselectivties. The high free volume of PIMs is due to the rigid backbone, in combination with a contorted structure, which results in an inefficient packing of the polymer segments in the condensed state.^25^ For the archetypal PIM, called PIM-1, the inefficient segment packing originates from a rigid ladder-like backbone in combination with sites of contortion in the repeating unit. In the bulk state, PIM-1 has interconnected pores with characteristic sizes less than 2 nm and large Brunauer–Emmett–Teller (BET) surface areas (>700 m^2^g^–1^).^26^

When a polymer is cooled from high temperatures to temperatures below the glass transition temperature (*T*_g_), several properties like enthalpy and entropy change in their temperature dependence around *T*_g_ resulting in a glass with a drastically reduced molecular mobility. The formed glassy solid, is in a thermodynamically nonequilibrium state. Therefore, at temperatures below *T*_g_ the polymer may slowly relax towards the thermodynamic equilibrium. This process is called physical aging and influences properties such as modulus, brittleness, and permeability.^27^ Physical aging has been associated with subsegmental relaxation processes such as reorientation of side groups, which are distinguished from cooperative fluctuations related to the glass transition. The rigid backbone structure of PIMs leads to a more premature solidification (e.g., by solvent evaporation during film casting) and a glassy state even farther from equilibrium. Therefore, compared to other glassy polymers, PIMs are even more prone to physical aging which leads to a decrease of their free volume.^28,29^ As a result, the aged membrane has less preformed pathways for gas molecules in a separation process, reducing the permeability and thus partly diminishing the favorable gas separation properties.

Different strategies have been developed to reduce physical aging, such as blending and cross-linking. Incorporating fillers into a polymeric matrix was found to be a successful approach to reduce this process. The obtained membranes are called mixed matrix membranes (MMM).^30,31^ The incorporation of a filler can alter the free volume in the polymer matrix and lead to a change in the molecular mobility, affecting physical aging as well as gas transport properties. Furthermore, if the interaction between the filler and matrix is strong enough, this can lead to a stabilization of the polymer matrix. An ideal MMM with an optimum performance should consist of well-dispersed filler particles at their maximum practical loading with an excellent filler/polymer contact.^30^

Several studies were reported focusing on fillers, which can be either accessible or inaccessible for the penetrant molecules, also providing insights into the polymer/filler interactions. Among them are porous Zr-based metal organic frameworks (MOFs),^32^ zeolitic imidazolate frameworks a subclass of MOFs,^33,34^ carbon nanotubes,^35^ polyhedral oligomeric silsesquioxanes (POSS),^36,37^ porous organic frameworks (POFs),^38^ and porous aromatic frameworks (PAFs)^39,40^ just to mention a few.

For MOFs and zeolites as porous fillers, despite improvements in the permeability values for some gases, the fillers have a weak interaction with the polymer matrix. Therefore, an aggregation of the MOF particles is often difficult to avoid^31^ and the resulting poor interface morphology may produce interfacial voids. On the other hand, a strong interaction may result in a rigidification of the polymer segments around the filler particles resulting in a reduced molecular mobility and penetrant diffusivity, and possibly to a blocking of the pores of an accessible filler. An optimal match between filler and matrix is therefore the prerequisite for a synergistic improvement of the separation performance for MMMs.^41^ Nevertheless, for some MOF-based MMMs it was shown that the organic compounds of MOFs can contribute to a better compatibility with the polymeric matrix. In contrast to inorganic–organic hybrid MOFs, organic based fillers such as covalent organic frameworks (COFs), PAFs, and POFs might have better potential in achieving a MMM morphology with fillers well distributed in the matrix and matching interfacial properties between the two materials.^30^

Here, a novel π-conjugated, phosphinine-based covalent organic framework is employed as a porous filler for the preparation of a MMM. By incorporating this filler in a PIM-1 matrix, it is anticipated to create additional gas transport channels or favorably modify the microporous morphology in the resulting MMMs to increase gas permeability and/or permselectivity as well as to reduce physical aging.

To understand the impact of nanofillers on the molecular dynamics of the polymer matrix, here, broadband dielectric spectroscopy (BDS) investigations are conducted in addition to gas transport measurements. The results of these measurements are comparatively discussed to establish a correlation between molecular mobility and gas transport properties.

## Experimental Section

### Materials

**Figure 1:**
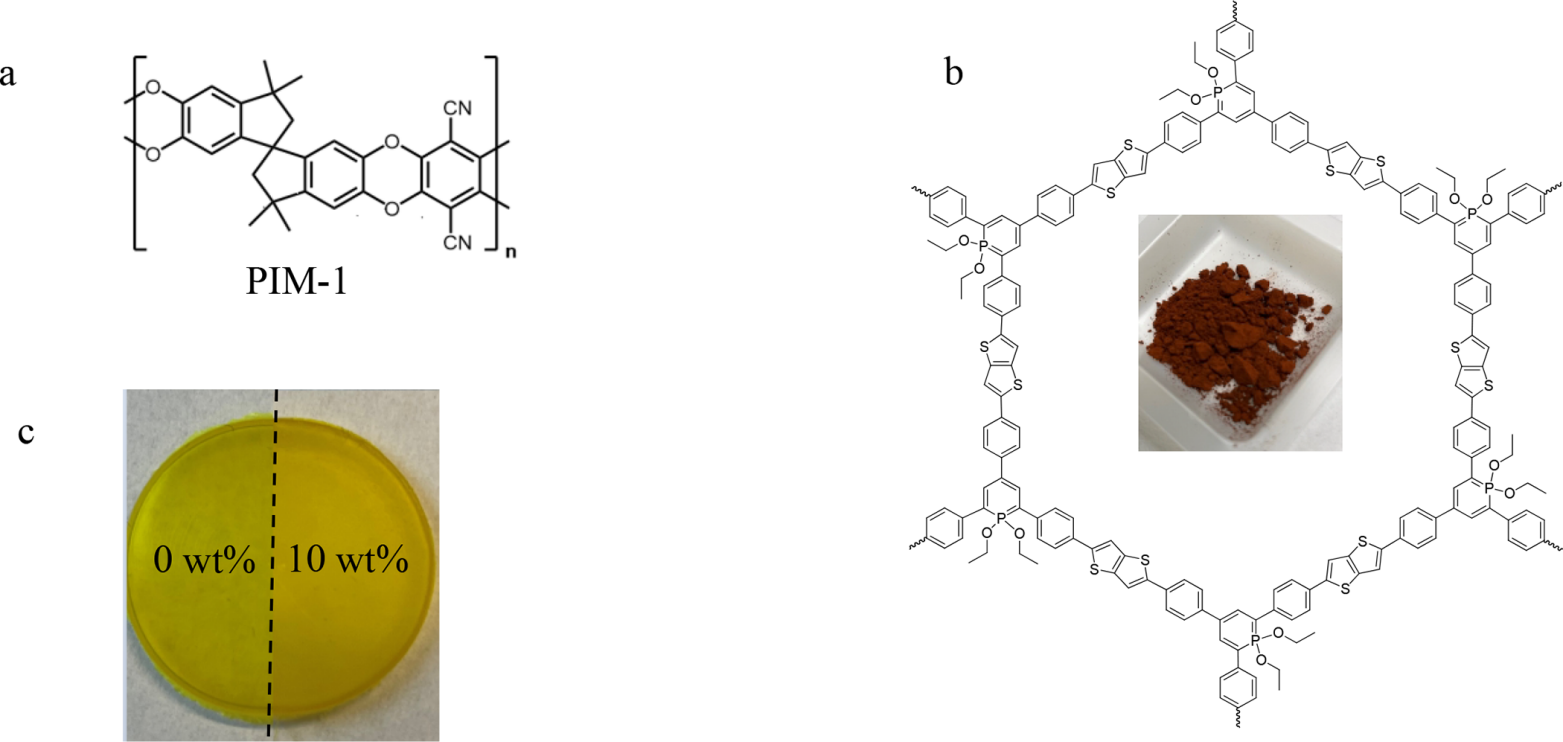
Chemical structure of (a) PIM-1 and (b) CPSF-EtO; (c) image of cast films for pure PIM-1 and the MMM with 10 wt % CPSF-EtO.

The synthesis of PIM-1 was carried out according to ref (42). For details, see the Supporting Information Scheme S1 and Figure S1. The chemical structure of PIM-1 is shown in Figure 1a. Size exclusion chromatography (SEC) was carried out using a Viscotek VE2001 system with two PL mixed B columns and a Viscotek TDA 302 multidetector array, taking chloroform as solvent. These experiments gave a weight-average molecular weight of Mw = 106200 g mol^–1^ and a polydispersity index (PDI) of 1.8 for the PIM-1 sample. Concerning synthesis and resulting backbone topology the PIM-1 discussed here corresponds to the sample B2 considered in refs (43,44). The FTIR spectrum of PIM-1 is given in the SI, Figure S2. In the bulk, PIM-1 has a microporous morphology, as shown by positron annihilation lifetime spectroscopy (PALS) and BET measurements. An average size of the micropores of 0.48 nm was found by PALS measurements assuming a monomodal distribution of spherical free volume elements.^45^ Considering a bimodal distribution, dimensions of 0.25 and 0.52 nm are obtained for the sizes of the pores.^46^ The BET surface area is found to be ca. 720 m^2^ g^–1^.^26^ It is worth mentioning that the pores form an interconnected network.

No glass transition temperature of PIM-1 could be estimated by conventional calorimetry before degradation of the polymer. However, by employing fast scanning calorimetry a glass transition temperature (*T*_g_) of 715 K at a heating rate of 30 kK s^–1^ was estimated for PIM-1.^47^ Notably, the shift of the glass transition with the heating rate was also investigated in this paper^47^ and later compared to a series of related polymers of intrinsic microporosity.^48^

The COF filler was synthesized from covalently linked π-conjugated building blocks.^49^ A Stille coupling reaction incorporating the six-membered phosphinine ring into a COF was employed, yielding a honeycomb-like aromatic structure. The novel covalent framework used in this study, based on λ^5^-phosphinine tectons also incorporating thienothiophene moieties, is denoted as CPSF-EtO and its chemical structure is depicted in Figure 1b. Some details about the synthesis can be found in the Supporting Information of ref (50). For instance, the elementary analysis shows an almost complete coupling reaction with a 2D polymer sheet formation without any collapse of the ring structure. Most likely two-dimensional sheets are formed during the synthesis, which stack together to larger aggregates. The difference between the covalent organic framework CPF-1 discussed in ref (49) and CPSF-EtO is that CPSF-EtO has the central phenyl rings replaced by sulfur containing thienothiophene units. The FTIR spectrum of CPSF-EtO is shown in Figure S2. CPSF-EtO shows a slight microporosity characterized by a BET surface area of 92.2 m^2^ g^–1^ where the average pore size is found to be in the range of 4 nm. CPSF-EtO bulk was investigated as powder by electron microscopy.^50^ The images show a flake like structure exhibiting a size distribution with an average value of ca. 37 μm. A close-up of the flakes reveals that they have a finer internal structure. Therefore, the pure filler was also investigated by X-ray scattering combining the SAXS and WAXS range.^50^ Average globular particle dimensions of about 80 nm were obtained from a Monte Carlo fitting analysis of the scattering in the SAXS region (McSAS). (For details of the Monte Carlo fitting analysis see ref (51).) These investigations confirm that the structure of CPSF-EtO is not crystalline but can be considered as an amorphous glass. It is discussed in detail in ref (49) that an amorphous structure is quite often observed for COFs prepared by a Stille coupling protocol.^49^ As reported in a separate paper,^50^ the pore diameter found experimentally corresponds well to the value that can be extracted from the chemical structure. However, the low value of the BET surface area of 92.2 m^2^ g^–1^ indicates that the stacking of the 2D sheets is predominantly irregular, causing an overall amorphous structure of CPSF-EtO. A more detailed discussion can be found in ref (50).

Furthermore, the pure filler was further investigated by fast scanning calorimetry (FSC) and broadband dielectric spectroscopy (BDS).^50^ Both methods show that CPSF-EtO undergoes a (dynamic) glass transition with an extrapolated glass transition temperature of 385 K at a heating rate of 1 kK s^–1^. One must be noted that conventional DSC measurements (10 K min^−1^) gave no clear signal. The dielectric spectra show a further process besides the dynamic glass transition. Its temperature position is independent of frequency. Therefore, this process is assigned to a percolation of electrical excitation, which is found also for other porous systems.^52,53^

### Film Preparation

Films of MMM were prepared by solution casting. The neat PIM-1 film was prepared by dissolving 0.1 g of PIM-1 in 3 mL of chloroform. For MMMs with the selected concentrations of the filler (2, 5, 7, and 10 wt % of CPSF-EtO), different amounts of CPSF-EtO powder were dissolved in 3.5 mL chloroform. The mixture was treated by ultrasound for 30 min followed by shaking for a further 30 min. This step was repeated two more times before the required amount of PIM-1 was added to the mixture as powder. Afterward, the polymer/filler mixture was stirred overnight. Then the obtained solution was filtered (2 μm PTFE- filter) and treated again by ultrasound for 5 min. After this procedure, the solution was cast into a Teflon mold (20 mm diameter). To slow down the evaporation of the solvent, the mold was placed in a closed chamber saturated with chloroform vapor for 72 h. Afterward, the formed films with a thickness of approximately 0.1 mm were removed from the mold and dried in an oil-free vacuum at 75 °C (348 K) for 72 h to remove the solvent as much as possible. It is discussed in the literature that an alcohol treatment might reduce the amount of entrapped solvent.^54^ Such an approach is not employed here because the influence of the alcohol on the morphology of the PIM-1 matrix and especially on the composites is not known. Moreover, it is further known that such an alcohol treatment not only leads to significantly higher gas permeabilities but also accelerates physical aging. Consequently, the observed gas permeabilities are in the lower range of values reported in the literature.^55^ The macroscopic morphology of the samples was characterized by optical photography (Figure 1c). The obtained films have a smooth surface. They are transparent but with increasing filler content, the films become more and more yellowish. The transparency of the samples indicates that there is no macroscopic phase separation on a length scale larger than half of the wavelength of visible light. The nanoscopic morphology was further investigated below by electron microscopy and X-ray scattering. The samples are denoted as PIM-1-X, where X refers to the formulated concentration of the filler. The FTIR spectra and thermal stability (TGA) of the composites are compared with that of PIM-1 and CPSF-EtO in Supporting Information, Figures S2 and S3. As for neat PIM-1, by conventional DSC no glass transition could be detected for the MMM before decomposition. For technical reasons no FSC investigations could be carried out for the prepared composites.

### High-Resolution Scanning Electron Microscopy (T-SEM)

High-resolution scanning electron microscopy in transmission mode (STEM-in-SEM, or T-SEM) has been carried out at a SEM of the type Supra 40 (Zeiss, Oberkochen, Germany) equipped with a Schottky-field emitter and a dedicated sample holder. It is able to provide transmission imaging, described in detail elsewhere.^56^ For this purpose, the samples have to be prepared in a similar way to conventional transmission electron microscopy (TEM), i.e., as electron-transparent specimens with thicknesses of about max 100 nm fixed on a TEM-grid. All samples in this study analyzed by STEM-in-SEM were prepared as thin slices by microtomy. Then, the samples were placed carefully on a carbon TEM grid. Hence, with the STEM-in-SEM option, it was possible to investigate the inner structure/morphology of the nanocomposites with a high imaging contrast. The applied accelerating voltage was 10 kV.

### X-Ray Scattering

The morphology of the MMM samples was further investigated by X-ray scattering employing the MOUSE instrument (Methodology Optimization for Ultrafine Structure Exploration).^57^ The basis of MOUSE is a Xeuss 2.0 instrument (Xenocs, Grenoble, France) that is highly customized. X-rays were generated from a cooper target with a Cu Kα wavelength of 0.1542 nm using a microfocus X-ray tube. Further, the X-ray beam is monochromatized and parallelized by multilayered optics. The scattered X-rays were detected with an Eiger 1 M detector (Dectris, Baden, Switzerland). The distance between the sample and detector was varied from 52 to 207 mm to allow for small- and wide-angle scattering (SAXA, WAXS). The software package DAWN was employed for the processing of the measured data.^58^ The data treatment is a complete, universal procedure that also propagates uncertainties.^59^

### Broadband Dielectric Spectroscopy (BDS)

The molecular mobility as a molecular probe for structure was studied by broadband dielectric spectroscopy (BDS). A high-resolution ALPHA analyzer connected to a sample holder with an active sample head (Novocontrol, Montabaur, Germany) was used to measure the complex dielectric permittivity ε*(*f*) = ε^′^(*f*) – *i*ε^″^(*f*) in the frequency range from 10^–1^ Hz to 10^6^ Hz (*f =* frequency, ε^′^ = real part, ε^″^ = loss or imaginary part, ) in parallel plate geometry.^60^ To ensure a good electrical contact between the sample and the electrodes of the sample holder (gold-coated brass electrodes, 10 mm in diameter), gold electrodes with a diameter of 10 mm were evaporated on both sides of the film. The measurements were carried out by isothermal frequency scans applying a temperature program with several heating and cooling cycles in the range of 173 to 523 K. A Quatro cryosystem (Novocontrol) was used to control the sample temperature, providing a temperature stability better than 0.1 K. During the whole measurement, the sample was kept in a dry nitrogen atmosphere.

### Gas Transport Measurements

The time-lag (TL) method was used to determine the gas transport properties for PIM-1 and the PIM-1/CPSF-EtO composites.^3^ An example of a measurement is depicted in Figure S4 in the Supporting Information. The polymer film was placed in a temperature-controlled permeation-cell on a porous support and sealed by a Viton O-ring. The effective area for gas transport was *A* = 1.33 cm^2^. Before the permeation measurement, the film was degassed at a pressure of 10^–6^ mbar for 72 h. The probed gas was fed into the upstream chamber with an upstream pressure *p*_1_. The employed pressure range was 1.0 to 10 bar. The downstream pressure *p*_2_ was measured in a closed downstream volume (previously evacuated) with a temperature controlled (100 °C) MKS Baratron gauge (type 628B, 10 mbar range).^61^

In the steady-state regime, *p*_2_ increases linearly with time. The permeability *P* was calculated from the slope of the linear increase in the steady state region according to^3,62^
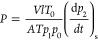
3where *V* is the downstream volume, *T* is the temperature, *l* is the sample thickness, *T*_0_ = 273.15 K, and *p*_0_= 1.013 bar. The permeability is given in Barrer units defined as
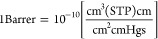
4

STP refers to standard conditions: temperature (273.15 K) and the pressure (1 bar = 10^5^ Pa).

An effective diffusion coefficient *D*_eff_ can be calculated from the time-lag τ and the film thickness l by
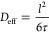
5

## Results and Discussion

### Morphology of the MMM

**Figure 2:**
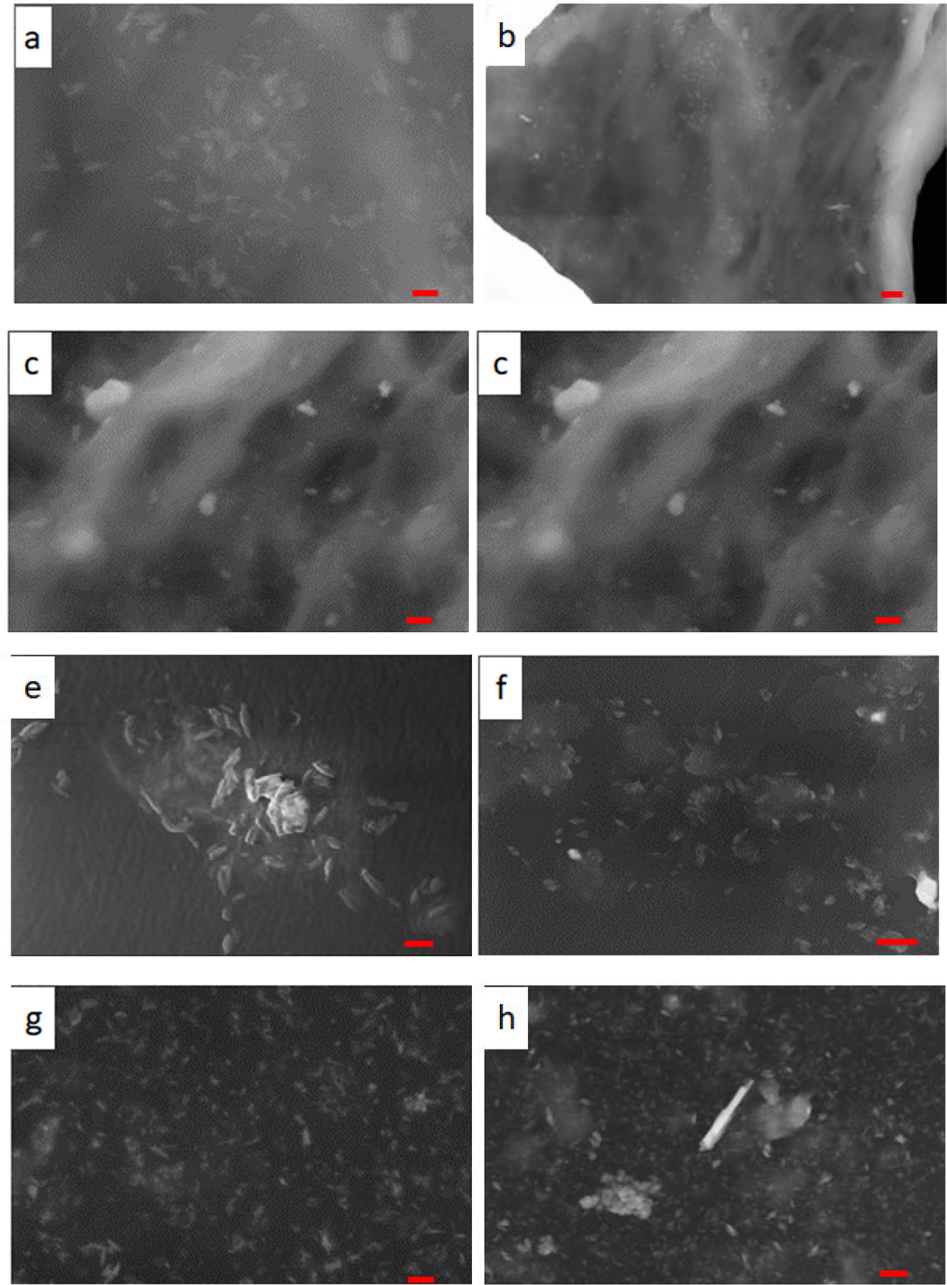
T-SEM images for PIM-1 (a,b), PIM-1–05 (c,d), PIM-1–07 (e,f), and PIM-1–10 (g,h). Red scale bar on the left side: 100 nm, scale bar on the right side: 300 nm (for PIM-1–10 200 nm).

The morphology of the prepared films was investigated by two different methods, allowing a characterization of the structure of the prepared composites on different spatial scales addressing primary particles (X-ray scattering) and larger agglomerates (electron microscopy). First high-resolution scanning electron microscopy (T-SEM) was employed with a resolution of 100 nm. Figure 2 gives exemplary T-SEM images for pure PIM-1 and the composites of three concentrations of CPSF-EtO at two magnifications. For pure PIM-1 and the composites some wrinkles are observed in the T-SEM images. Most probably they may result from the sample preparation as such wrinkles are also observed for the composites. This issue is further discussed in the context of the X-ray data. First, for the composites no flakes corresponding to the pure CPSF-EtO (where an average size of 37 μm was observed) were found. This means that the flake-like aggregates were successfully disrupted and dispersed during the preparation step. Second, the images show well distributed small aggregates or particles with a size up to ca. 100 nm. Some of these particles are even larger than 100 nm. These small aggregates might remain from the preparation step as the flakes of CPSF-EtO consisted of finer primary particles with an average size of 80 nm, which could not completely be broken by applying ultrasound to the dispersion. It is worth mentioning that these small particles could not be filtered out as the pores of the filter used prior to solution casting were larger. Nevertheless, the size of the primary particles is in the range up to 100 nm, which means that the prepared membranes can still be considered as nanocomposites. This observation does not rule out that CPSF-EtO might be incorporated (i.e., dissolved) in the MMM also as isolated sheets. Thus, for the prepared membranes, it is assumed that the CPSF-EtO filler is incorporated both as isolated sheets as well as in the form of small primary aggregates with a size up to ca. 100 nm. For higher concentrations of the filler, the primary aggregates can partly agglomerate to larger secondary domains (see Figure 2e or h).

**Figure 3:**
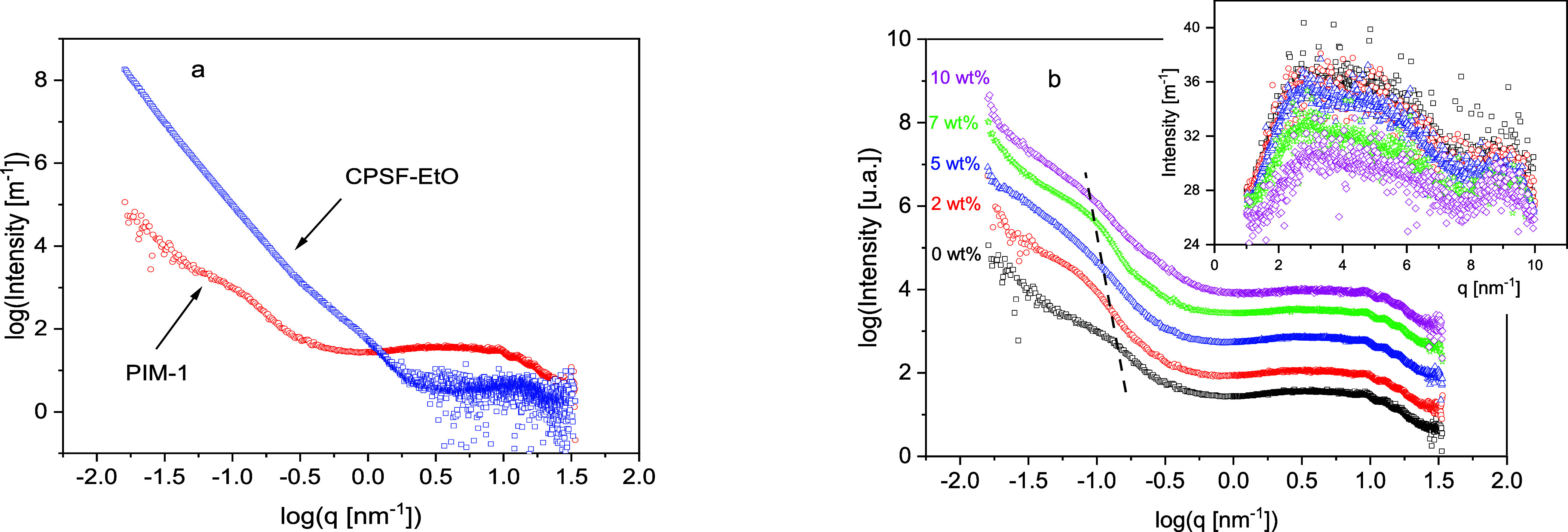
(a) X-ray pattern for PIM-1 (red circles) and CPSF-EtO (blue squares). (b) X-ray pattern for PIM-1 and the composites as indicated. The curves are shifted along the y scale for the sake of clarity. The inset gives the scattering in the q range from 1 to 10 nm^–1^.

Figure 3 compares the X-ray scattering pattern for pure PIM-1 and CPSF-EtO. The scattering data for pure PIM-1 have been already discussed elsewhere.^63,64^ In principle, the scattering pattern obtained here for PIM-1 is similar to that discussed in ref (63). Nevertheless, it is worth noting that the samples were prepared in different ways. For q vectors ≥8 nm^–1^, a broad scattering event is observed which consists of at least 3 peaks. First, simulations provide some evidence that these peaks in the high q range might be related to a characteristic distance between spiro centers of repeating units of different chains.^63^ Alternatively, one can consider that these peaks originate from a structure factor related to microporosity and an underlying form factor.

It was further argued that the scattering in the q-range of 2 to 8 nm^–1^ is somehow related to the microporosity. Nevertheless, it was discussed already in ref (63) that it is somehow ambitious to estimate pore sizes from this region. Such a procedure would lead to pore dimensions greater than 1 nm. As discussed above, other techniques (such as PALS) gave pore dimensions smaller than 1 nm.

As a second method to characterize the morphology of the samples, X-ray scattering was applied with a resolution from Angstroms up to 100 nm. In the SAXS range, a structural feature is observed at ca. 0.1 nm^–1^. The data in the q range from 10^–2^ to 1 nm^–1^ was analyzed by a Monte Carlo fitting analysis taking a sphere as scattering object. This analysis gives a dimension of 14 nm. The molecular origin of this scattering is probably a supramolecular structure of PIM-1 formed during the preparation of the membranes. It is worth mentioning that the size of these structural features is smaller than the size of the wrinkles seen in the T-SEM images (Figure 2a,b).

The X-ray patterns for PIM-1 and the composites are given in Figure 3b. For the composites, also a scattering feature is observed in the SAXS region at similar q values as for pure PIM-1. With increasing filler concentration, this feature seems to shift to smaller q values indicating a scattering from larger objects. Figure 3a compares the scattering of pure PIM-1 with that of pure CPSF-EtO. This figure reveals together with Figure 3b that in contrast to other systems (see for instance ref (65)) the scattering behavior of the PIM-1/CPSF-EtO is not directly additive. This supports the observation that the aggregates with an average size of ca. 80 nm found for pure CPSF-EtO are further disrupted into smaller structures during the preparation of the composite in the dispersion step by applying ultrasound. This conclusion was drawn from the T-SEM images, which also show small aggregates with sizes up to 100 nm in the composites.

The observed scattering of the composites is the superposition of the scattering originating from the PIM-1 matrix and from the primary aggregates of CPSF-EtO. As for PIM-1, the X-ray data for the composites were analyzed by a Monte Carlo fitting procedure (q range 10^–2^ to 1 nm^–1^) assuming spheres as scattering objects. This analysis reveals that the mean size of the scattering objects shifts from 14 nm observed for PIM-1 to 62 nm for the composite with 10 wt % CPSF-EtO. From the Monte Carlo analysis also the size distribution of the scattering object is obtained. The corresponding histograms are given in the Supporting Information (Figures S5–S8). These histograms show that for the composites, the scattering objects have a broad distribution which shifts to larger distances with increasing concentration of the filler. These results are consistent with the observations in the corresponding T-SEM images. For the highest concentrations of CPSF-EtO, there is an upturn in the scattering intensity for the lowest q vectors. This upturn points to scattering coming from even larger objects.

The inset of Figure 3b enlarges the observed scattering pattern in the q range from 1 to 10 nm^–1^. As discussed above, this region is sensitive to the microporous structure. The intensity of the scattering in this region decreases systematically with an increasing concentration of the filler. This means that the structure of the formed microporosity is influenced by the presence of the incorporated filler. In the context of investigations addressing structure and dynamic of microporous composite systems, it has to be noted that this is fundamentally different from impregnating a polymer or a soft matter system into a microporous system like sol–gel glasses or anodic aluminum oxide (see for instance refs (66,67)). Here, the fixed and rigid microporous structure of the host matrix (acting as a confining outer network) is modified by the soft filler.

From the gradual decrease of the scattering intensity with increasing filler concentration (especially for 7 and 10 wt %) observed for the polymer composites under investigation here, it can be concluded that the presence of the nanoscale filler and its aggregates and agglomerates changes the structure of the microporosity formed during solidification of the dissolved PIM-1 by solvent evaporation.

### Broadband Dielectric Spectroscopy

BDS is sensitive to fluctuations of permanent dipoles related to the chemical structure. The dielectric behavior of pure CPSF-EtO was discussed in detail in ref (50) and in brief in the materials section above. The dielectric relaxation behavior of pure PIM-1 is discussed in detail elsewhere.^29^ As in previous studies, the BDS measurements of the PIM-1 materials in this study show a substantial change in the dielectric spectra for the first compared to the second heating run, while the subsequent cycles are similar to the second heating cycle. Thus, here, the dielectric spectra obtained for the second heating run are further discussed.

**Figure 4:**
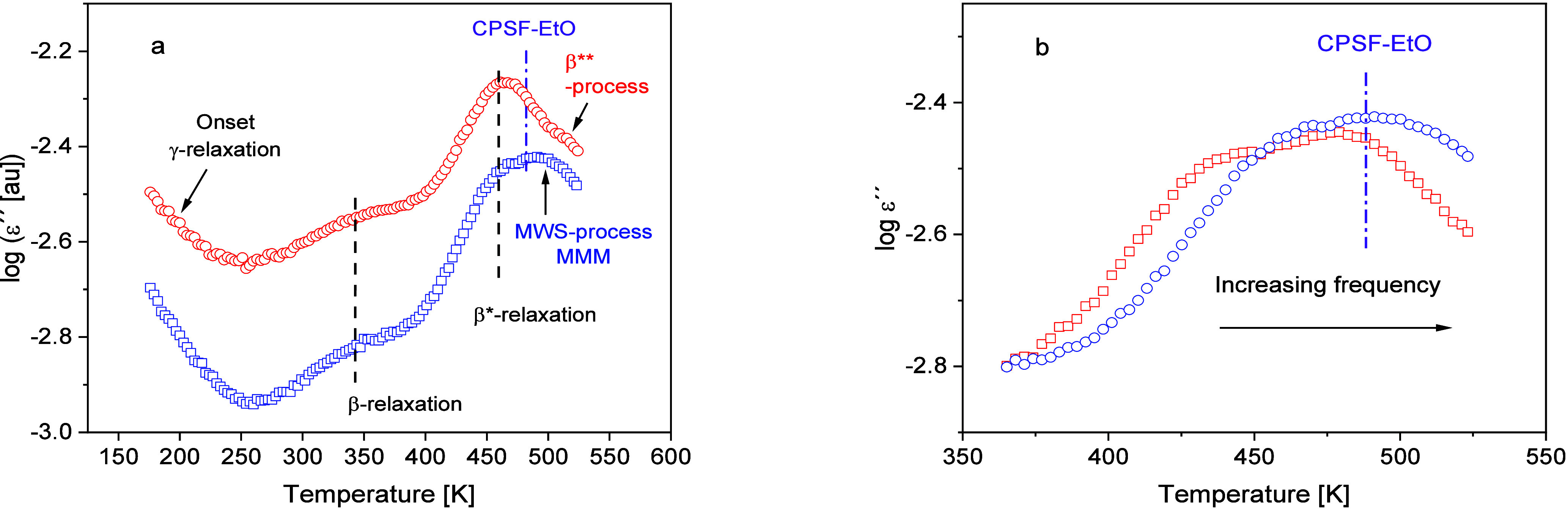
(a) Dielectric loss versus temperature at a fixed frequency of 1000 Hz for pure PIM-1 (red circles) and the MMM with 5 wt % CPSF-EtO (blue squares). The dielectric loss is shifted along the y-scale for the sake of clarity. The dashed line indicates the β- and β*-relaxation observed for PIM-1 as well as for PIM-1–05. The β**-process observed for PIM-1 is indicated in red. The MWS process observed for the MMM is indicated in blue. The violet dashed dotted line indicates the temperature position of the electrical percolation process observed for pure CPSF-EtO, for which temperature position is independent of frequency (see ref (50)). (b) Dielectric loss versus temperature for the MMM with 5 wt % CPSF-EtO in the temperature range of the MWS polarization for different frequencies: Red squares: 315 Hz and blue circles: 1000 Hz. The violet dashed dotted line indicates the temperature position of the electrical percolation process observed for CPSF-EtO, for which the temperature position is independent of frequency.

Several dielectrically active processes are observed for neat PIM-1 (Figure 4a). At low temperatures or high frequencies, a so-called γ-relaxation is found related to methyl group rotation but also involving polar units.^68^ A β-relaxation is observed at higher temperatures related to fluctuations of agglomerates of aromatic moieties as discussed in ref (36). A further process denoted as β*-relaxation is found at even higher temperatures. It was concluded that this process has in principle a similar origin as the β-relaxation.^29^ This similarity was discussed in terms of a spatial heterogeneity of the system. Finally, a further process is observed at elevated temperatures above 473 K (200 °C), denoted as β**-process. This process was attributed to an interfacial or Maxwell–Wagner–Sillars (MWS) polarization due to a blocking of charge carriers at internal pore walls.^29^

As observed for neat PIM-1, at least four dielectrically active processes are also detected for the MMMs. This is shown in Figure 4a where the dielectric loss versus temperature at a frequency of 1000 Hz is compared for pure PIM-1 and the MMM with 5 wt % CPSF-EtO as an example. All dielectric relaxation processes that are present for pure PIM-1 are also observed for the composites. However, the comparison also reveals some differences. First, the dielectric loss seems to be lower for the composites than for pure PIM-1. The reason might be the weaker molecular dipole moment of CPSF-EtO, which reduces the overall dipole moment of the composite compared to that of pure PIM-1. Second, in the temperature range of the β*-relaxation the observed peak is bimodal. One of the processes underlying the bimodal peak structure coincides in its temperature position with that of the β*-relaxation found for pure PIM-1. Therefore, also for the composites, this process is assigned to the β*-relaxation with a similar underlying molecular mechanism as for pure PIM-1. The second process observed for the MMM is shifted to higher temperatures compared with that of this β*-relaxation. At the first glance, it could be argued that this additional peak observed for the MMM is due to CPSF-EtO. However, it was found that the temperature position of the electrical percolation process observed for pure CPSF-EtO is independent of frequency. For details, see ref (50). To visualize this, the fixed position of this process of CPSF-EtO is added as a vertical line to Figure 4a. This is different for the additional process in the composite materials, where the peak shifts to higher temperatures with increasing frequency (see Figure 4b). For that reason, this process should not be assigned to the percolation process observed for pure CPSF-EtO. As the β**-process observed for pure PIM-1 is not found for the composites, it is likely that the new process observed for the composite is an MWS process like that in PIM-1 but modified by the filler particles. It is worth noting that none of the dielectric processes observed for the pure filler can be found in the dielectric spectra of the composites.

**Figure 5:**
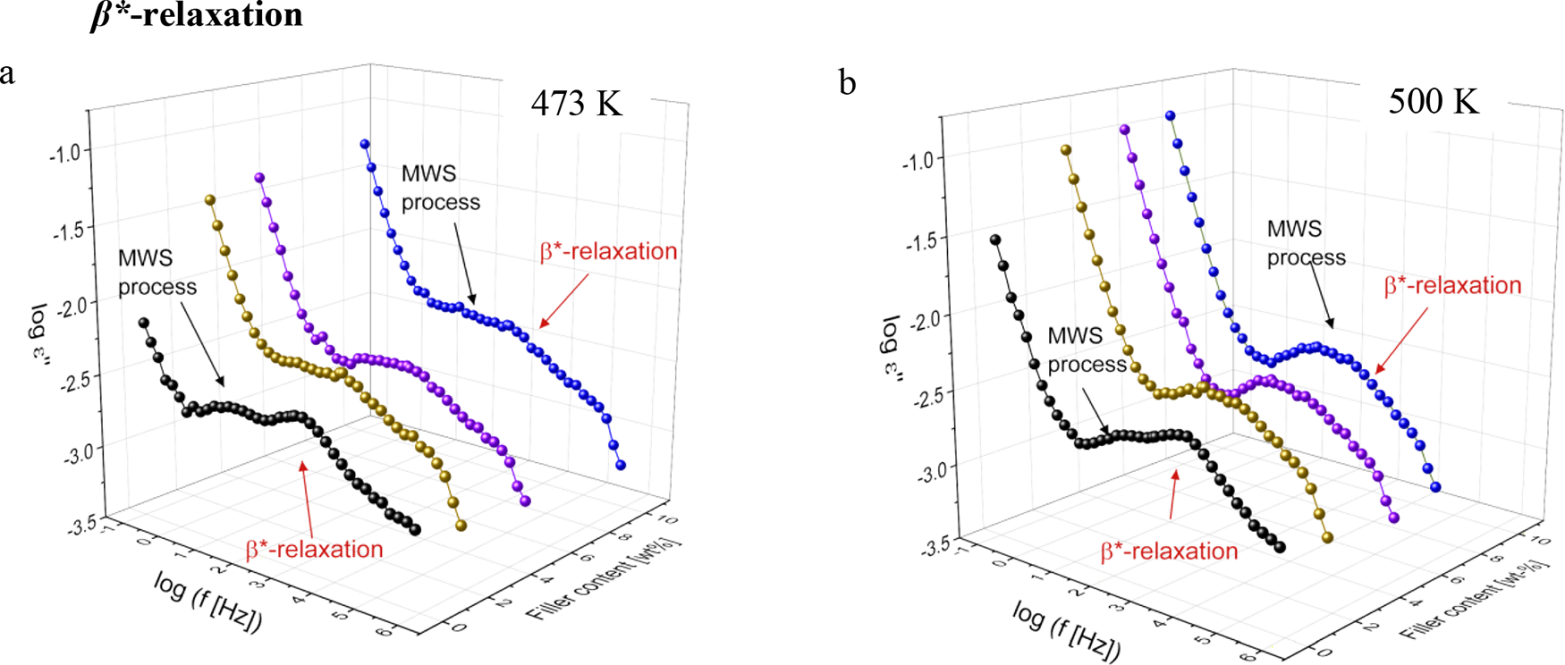
Dielectric loss spectra vs frequency and concentration of Eto-CPSF filler in second heating at (a) 473 and (b) 500 K for the investigated MMMs. The corresponding peaks for β*-relaxation and MWS process are indicated by black and red arrows, respectively.

The dielectric loss is plotted versus frequency and concentration of CPSF-EtO at *T* = 473 and *T* = 500 K in 3D representations for the investigated composites in Figure 5. There are several notable features: (1) there is an increase in the broadening of the β*-relaxation for all filled membranes (MMM) compared to PIM-1. This broadening could be explained by a larger heterogeneity induced by the filler. (2) At higher temperatures, the β*-relaxation and the MWS process merge together into a broad peak. This is due to a different temperature dependence of the characteristic rates of both processes as discussed below. (3) MMMs exhibit a higher conductivity contribution, which could be attributed to the presence of low concentration impurities associated with the added fillers.^69^ It might be possible that the sulfur atoms of CPSF-EtO also enhance the conductivity.

### β*-Relaxation

For further analysis of the dielectric relaxation processes, the model function of Havriliak–Negami^70^ (HN-function) was fitted to the dielectric data of the β*-relaxation. The HN function reads
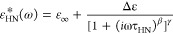
6Here, ω is the angular frequency, ω = 2π*f*. ε_∞_ represents the real part ε^′^ in the limit ε_∞_ = lim_ω≫τ_HN_^–1^_ε^′^(ω), Δε denotes the dielectric strength, and τ_HN_ is a relaxation time corresponding to the frequency of maximal dielectric loss *f*_*max*_ (relaxation rate). β and γ (0 < β, βγ ≤ 1) are shape parameters, which describe the symmetric and asymmetric broadening of the relaxation time spectrum with respect to the Debye function.^60^ An example of the analysis of the data by the HN-function is given in Figure S9. By that analysis the relaxation rate *f*_max_ is obtained in dependence of temperature.

**Figure 6:**
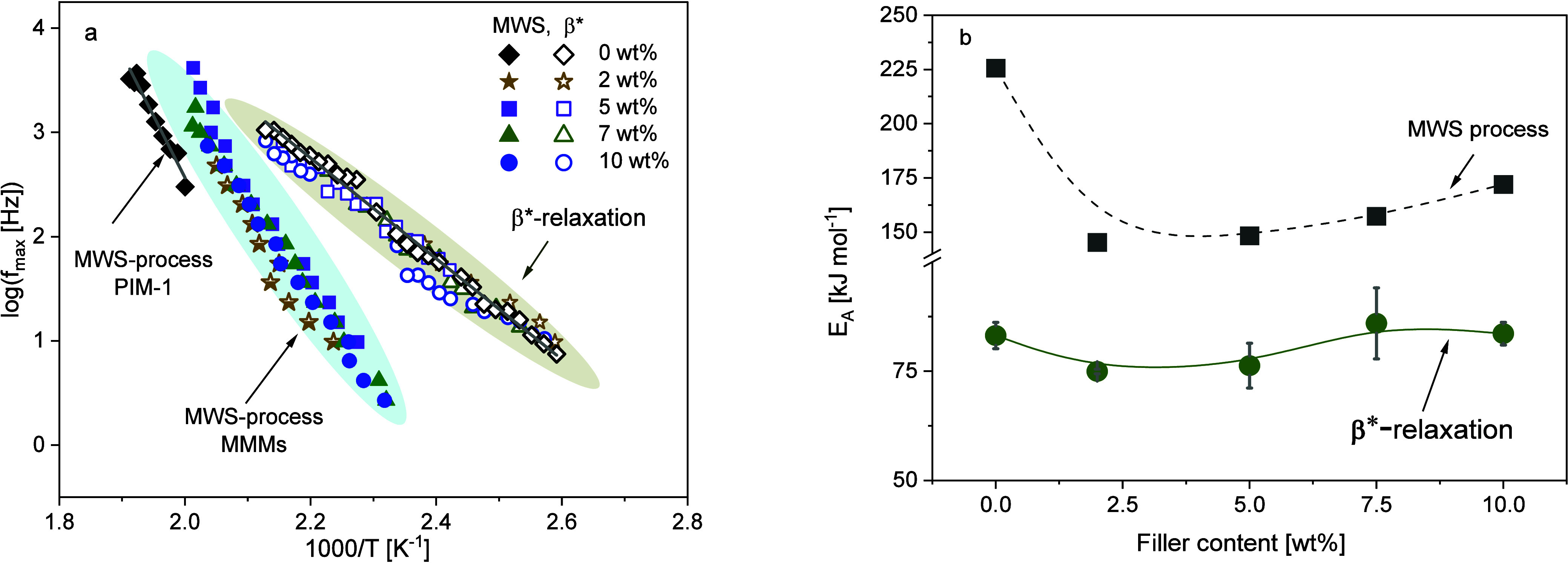
(a) Relaxation rate *f*_max_ for pure PIM-1 (black diamond) of PIM-1 with 2 wt % CPSF-EtO (PIM-1–02, brown star), 5 wt % CPSF-EtO (PIM-1–05, violet square), and 10 wt % CPSF-EtO (PIM-1–10, blue circles) for the second heating run. The gray line is fit of the Arrhenius equation to the data. (b) Activation energies for the second heating cycle vs the CPSF-EtO concentration: Green circles – β**-relaxation, black squares – MWS-process. The dashed and solid lines are guides to the eyes.

Figure 6a shows the temperature dependence of *f*_max_ for the β*-relaxation in the Arrhenius coordinates for all materials under investigation. The temperature dependence of the relaxation rate of the β*-relaxation obeys the Arrhenius equation, which reads:
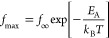
7Here, *f*_∞_ is a pre-exponential factor, *k*_B_ the Boltzmann constant, and *E*_*A*_ denotes the (apparent) activation energy.

As discussed earlier, the activation energy of the β*-relaxation for pure PIM-1is found to be ca. 83 kJ mol^–1^.^36^ This relatively high value points at least to a coordinated mechanism for the β*-relaxation. The corresponding activation energies for the MMMs are quite like that of the unfilled polymer (see Figure 6b). For low concentrations of the filler, there might be a slight decrease of the activation energy, which is probably due to a distortion of π–π aggregates of PIM-1 caused by the filler particles. Moreover, for higher concentrations of CPSF-EtO, there might be a small increase in the activation energy. This result can be understood considering a hindrance of the fluctuations of the agglomerates by the filler particles and/or that the free volume sites of PIM-1 are partly filled by CPSF-EtO. Furthermore, for the composites, the β*-relaxation shifts slightly to lower frequencies also for higher concentrations of the filler.

### MWS or Interfacial Polarization

The MWS process for MMM was also analyzed by fitting the HN-function to the data. Figure 6a also includes the frequency of maximal loss *f*_max_ for the MWS process in the activation diagram. Compared to pure PIM-1, the rates of the MWS process are shifted to lower temperatures for all MMMs. The rates of the MWS process seem to be located in a narrow frequency–temperature range, and the temperature dependence of this process obeys the Arrhenius law for all composites. The determined activation energies for all samples are given as a function of the CPSF-EtO concentration in Figure 6b. First, the activation energies obtained for the composites are significantly lower than those of pure PIM-1. Second, for the composites the activation energy increases approximately linearly with increasing CPSF-EtO concentration.

For PIM-1 the β**-process is due to blocking of charge carriers at the pore walls. In the considered composite system, the Maxwell/Wagner/Sillars polarization effect can generally have two different origins: the blocking of the charge carriers at nanofiller/polymer interfaces and/or at pore walls within the polymer matrix. The activation energy of the MWS process for the composites is found to be lower than for pure PIM-1 and to increase linearly with the filler concentration. Therefore, it is reasonable to assign the observed polarization process to interfacial polarization effects involving the CPSF-EtO filler. As shown by the T-SEM images and the X-ray data discussed above, at least a fraction of the filler particles for the composites is organized in small aggregates with sizes up to ca. 100 nm. Thus, a blocking of charge carriers at the aggregate/matrix interface seems to be likely. This process is denoted as MWS_filler_. Nevertheless, blocking of the charge carriers at the pore walls of PIM-1 and that of the CPSF-EtO filler could not be completely ruled out completely (MWS_pore_). In fact, the observation that the presence of the filler clearly alters the structure of the microporosity of the PIM-1- matrix would rather lead to the assumption that a combination of both effects is observed. Keeping this in mind, one should expect that the MWS process observed for the composites should be at least bimodal due to the two underlying processes MWS_filler_ and MWS_pore_. A further analysis of the relaxation spectra in the temperature range of the β*-relaxation and the MWS process reveals that for the composites the MWS process is broader than the β**-process for PIM-1. This might indicate that the MWS process for the composites is actually at least bimodal (see Figure 5).

Generally, for polymer-based composites, an interfacial polarization is observed only at temperatures higher than the glass transition temperature of the matrix. This is because in conventional polymers, the mobility of charge carriers is coupled to the segmental dynamics, which only can take place above *T*_g_. Therefore, the transport of the charge carriers becomes active only above *T*_g_ and their blocking at interfaces can cause such an interfacial polarization process. However, for microporous polymers, another conduction mechanism is considered. Here, the charge transport is rather due to the transport of mobile charge carriers through the interconnected network of micropores.^36^ First, such a conduction mechanism can be considered for MWS_pore_. Second, as segmental fluctuations are excluded because the polymer matrix is far below the glass transition temperature *T*_g_, a conduction mechanism related to a segmental mobility could be ruled out also for MWS_filler_. Therefore, it is assumed here that the incorporation of the filler creates additional free volume elements at the interface of CPSF-EtO aggregates to the PIM-1 matrix, which are connected to the microporous network. The conduction mechanism for MWS_filler_ in this model is diffusion of charge carriers through these additional free volume elements. In principle the increase of the free volume elements could be evidenced by BET surface area measurements, but as a larger sample amount is required, such measurements were difficult to carry out.

**Figure 7:**
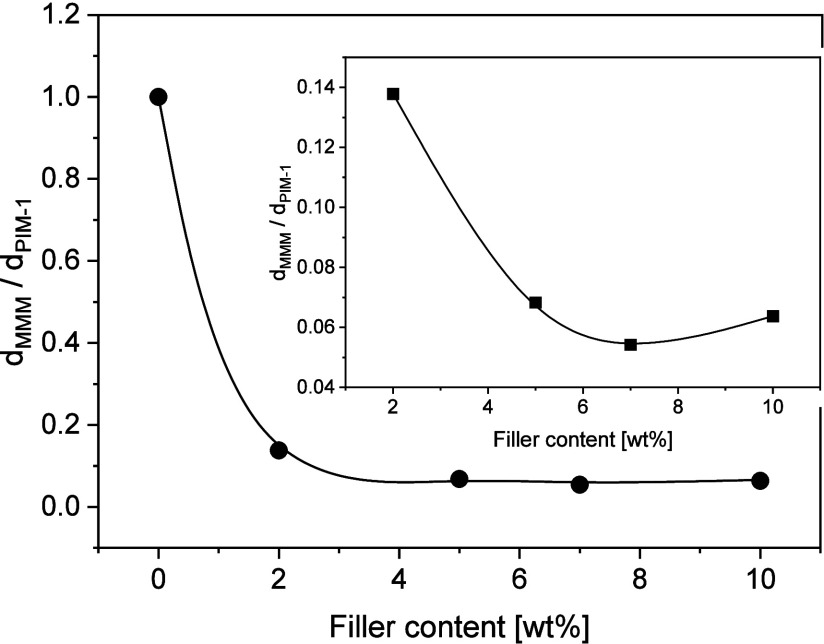
Ratio *d*_MMM_/*d*_PIM-1_versus the concentration of the filler at *T* = 462 K. The value for PIM-1 was calculated from the Arrhenius dependence of the rates of the β**-process. The insets enlarge the concentration range for the MMM composites.

An analysis of the MWS processes could provide further insights into the composite structure. The characteristic time constant of the MWS process can be discussed in a simplified model. In the alternating electric field, the charge carriers must move over a distance *d* before they are blocked by the phase boundaries. *d* corresponds to a mean distance between blocking interfaces. The blocking interface itself is described by an electrical double layer with an effective spacing characterized by its Debye length *L*_*D*_. The distance *d* is related to an additional capacitance in the system due to the charging and discharging of that electrical double layer. The time constant τ_MWS_ for the process can be estimated in the simplest possible approach by^60^
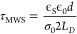
8where ϵ_0_ is the dielectric permittivity of vacuum and σ_0_ the DC conductivity of the system. Thus, the time constant is proportional to the mean distance *d* between the filler aggregates or pore walls. Because several quantities are not known, such as the thickness of the Debye layer, the absolute value of the average distance of the blocking interfaces *d* cannot be calculated. As an approximation, the following ratio
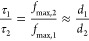
9can be considered to extract indirect information related to the observed changes in the distance of the blocking interfaces. The ratio d_MMM_/d_PIM-1_ is estimated from the data presented in Figure 6a and represents the changes of the characteristic distance of the blocking interfaces of the MWS process related to the overall microporosity of the PIM-1 matrix, i.e., pure PIM-1 and PIM-1 with different filler concentrations giving rise to the antagonistic effects of pore blocking and additional free volume at the filler interface as discussed below. It is plotted versus the concentration of CPSF-EtO in Figure 7 for the temperature *T* = 462 K. Such a plot might provide a rough estimate of whether the effective pore size is affected by the filler and/or how the distance between the filler aggregates is changed. The ratio *d*_MMM_/*d*_PIM-1_ decreases strongly from pure PIM-1 to PIM-1–02. Because the filler concentration is low and therefore the effect of the MWS_filler_ process should not be that pronounced as for higher concentrations, this result seems to indicate that the diffusion pathways of the charge carriers in the interconnected pore network are also decreased strongly in addition to the blocking of the charge carriers at the interface between the filler aggregates/PIM-1 matrix. This strong decrease in the diffusion pathways of the charge carriers might be due to a closing of bottlenecks of the interconnected pore network by isolated CPSF-EtO sheets as also evidenced by the X-ray data. This process results in a decrease in the effective distance of blocking interfaces. As the concentration of the filler is low the decrease in the diffusion pathways is not compensated by the creation of new free volume elements. The inset of Figure 7 enlarges the behavior of *d*_MMM_/*d*_PIM-1_ in the concentration range of the MMM. With increasing concentration of CPSF-EtO the ratio decreases further, indicating the stronger influence of the MWS_filler_ process. For the highest concentration, an increase of *d*_MMM_/*d*_PIM-1_ is observed due to an increase of the characteristic distance related to the pore size. This increase of the ratio *d*_MMM_/*d*_PIM-1_ indicates a decrease in the diffusion pathway of the charge carriers, which is due to a further aggregation of the aggregates to larger domains (see Figure 2e). These results will be further discussed together with the data obtained for gas transport in the next section.

### Gas Transport

The influence of the molecular topology of PIM-1 on its gas transport properties is discussed in detail in ref (44). As the pure PIM-1 is highly distributed in their molecular weight, this leads to a lower CO_2_/N_2_ selectivity than more highly branched PIM-1 samples. Furthermore, the samples investigated here were not subjected to an alcohol treatment (as it is discussed above). As such, a treatment leads to significant increases of gas permeability and is widely used in studies reporting permeability values of PIM-1 and related polymers the values obtained in the present work generally range in the lower limit of values reported in literature. Nevertheless, the similar preparation procedure of all PIM-1-based samples investigated in this study should ensure as far as possible similar states of aging and therefore still reflect the immediate changes related to effects of the filler in the most representative way. The gas transport properties of PIM-1 and selected composites were investigated by gas permeability measurements at 308 K in the upstream pressure range of 1 to 10 bar. Four gases N_2_ (3.64 Ȧ), O_2_ (3.46 Ȧ), CH_4_ (3.8 Ȧ), and CO_2_ (3.3 Ȧ) having different kinetic diameters given in the brackets were used.

### Permeability and Diffusion Coefficient

**Figure 8:**
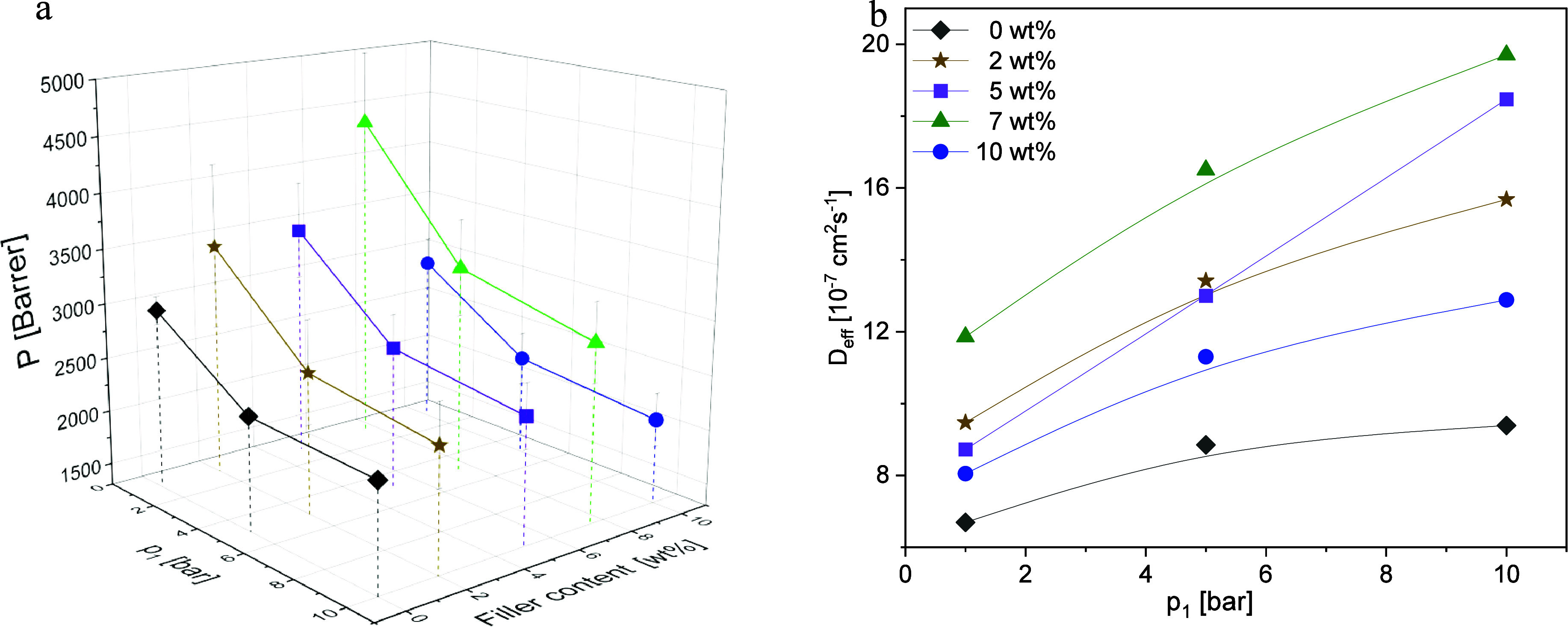
CO_2_: (a) Permeability vs upstream pressure *p*_1_ and filler concentration and (b) diffusion coefficients vs upstream pressure *p*_1_ at the indicated CPSF-EtO concentrations at *T* = 308 K. Lines are guides for the eyes.

**Figure 9:**
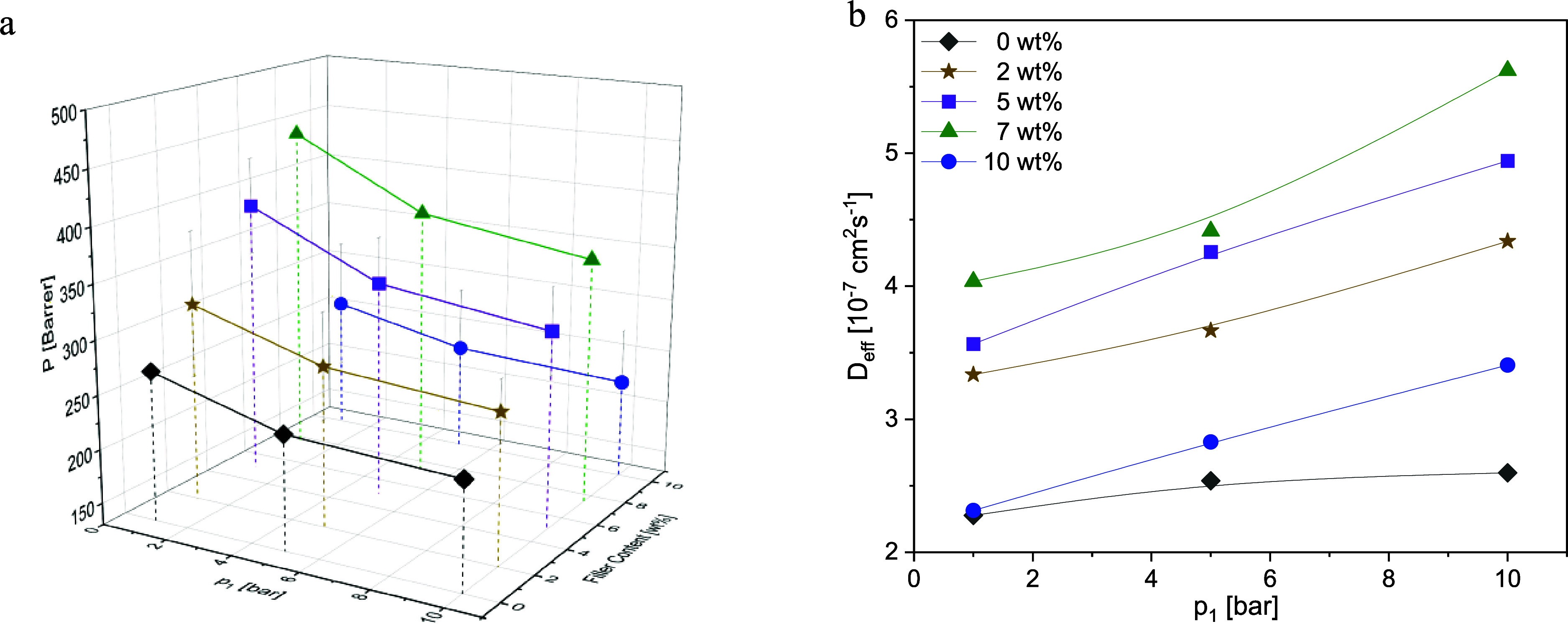
CH_4_: (a) permeability vs upstream pressure *p*_1_ and concentration and (b) diffusion coefficients vs upstream pressure *p*_1_ at the indicated CPSF-EtO concentrations at *T* = 308 K for the investigated composites. Lines are guides for the eyes.

**Figure 10:**
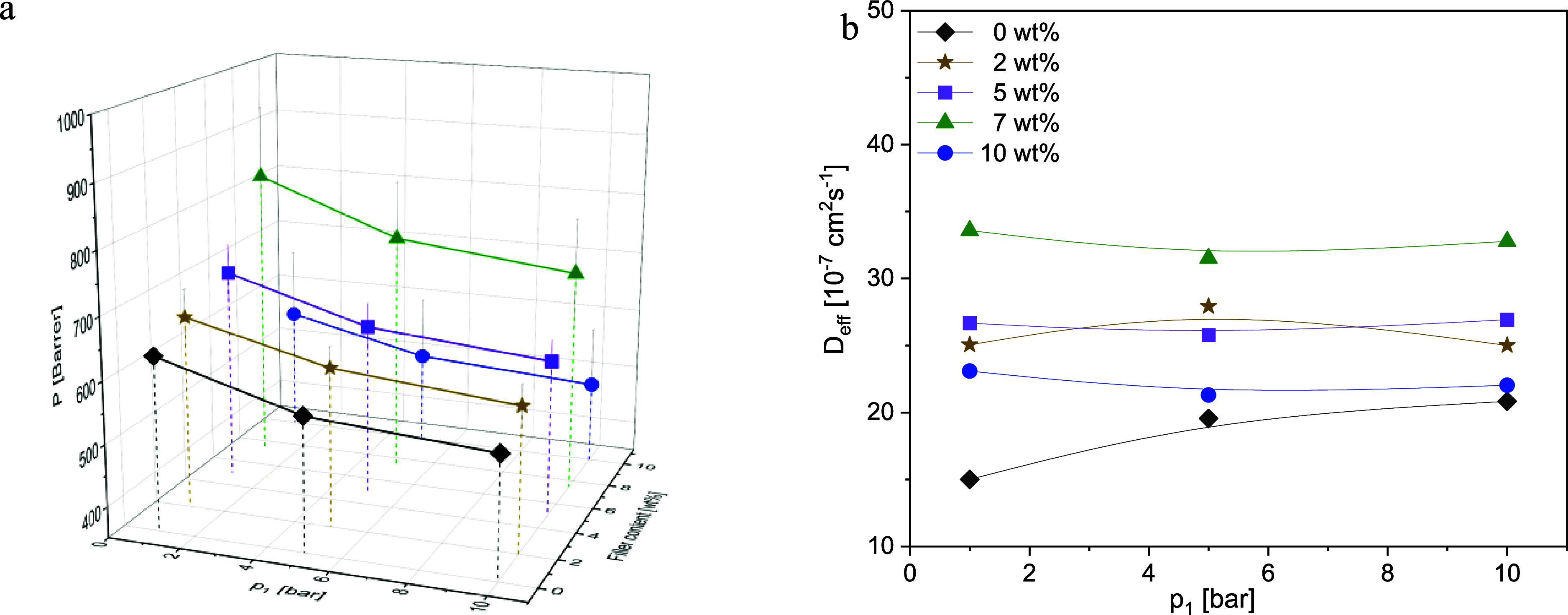
O_2_: (a) permeability vs upstream pressure *p*_1_ and concentration and (b) diffusion coefficients vs upstream pressure *p*_1_ at the indicated CPSF-EtO concentrations at *T* = 308 K for the investigated composites. Lines are guides for the eyes.

**Figure 11:**
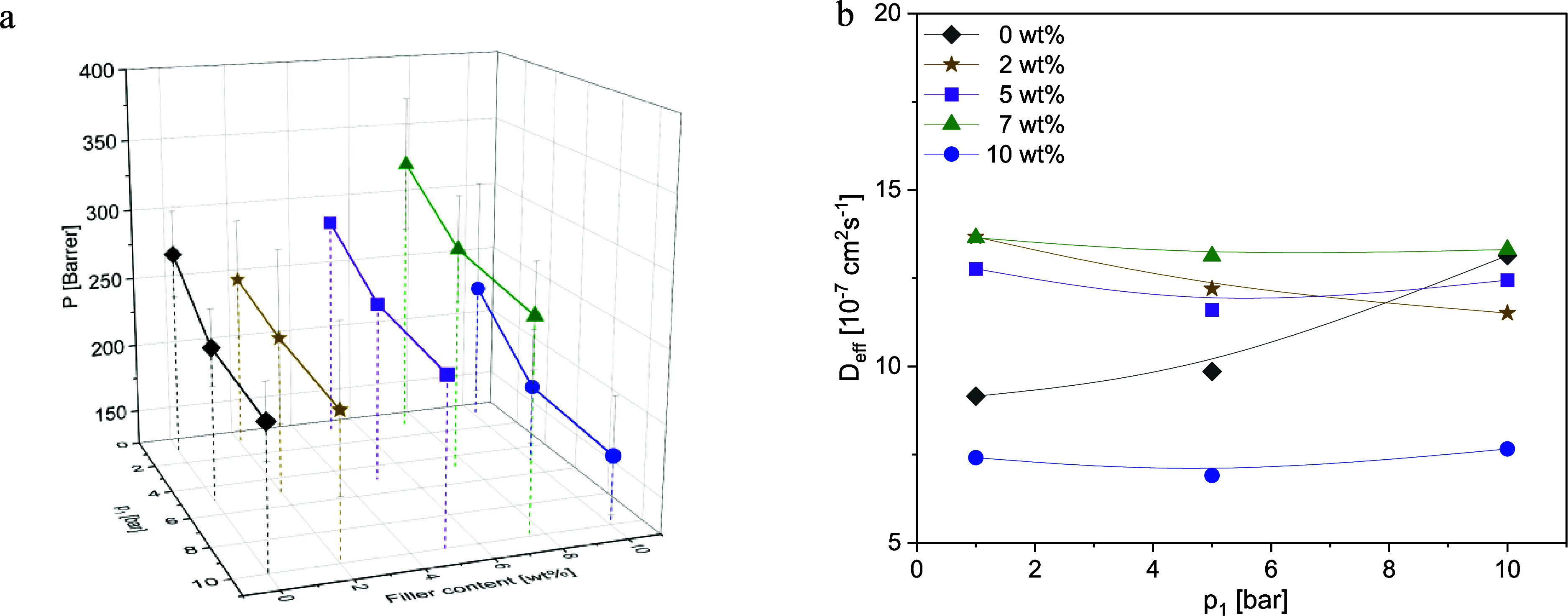
N_2_: (a) permeability vs upstream pressure *p*_1_ and concentration and (b) diffusion coefficients vs upstream pressure *p*_1_ at the indicated CPSF-EtO concentrations at *T* = 308 K for the investigated composites. Lines are guides for the eyes.

The gas permeabilities versus upstream pressure p_1_ and the concentration of the filler is plotted for *T* = 308 K as 3D graphs in Figure 8a to Figure 11a for all gases. For all MMM samples, the permeability values for the different gases at 5 bar are ranked as *P*_CO_2__ > *P*_O_2__ > *P*_CH_4__ > *P*_*N_2_*_ as shown in Figure S10 in the SI. The effect of the CPSF-EtO concentration on the diffusion coefficients of all used gases versus upstream pressure is shown in Figures 8b–11b.

For polymers in the glassy state pressure dependent sorption and permeation are generally expected, often described by the dual-mode sorption model.^71^ Primary basis is the decreasing gas solubility with increasing pressure, which in most cases also leads to a decreasing gas permeability (cf. eq 1). With respect to the diffusivity different behavior may occur. For strongly sorbing gases, such as CO_2_ or CH_4_, often a plasticizing effect is observed at elevated pressures (and higher gas loadings), resulting in an increased mobility of the matrix and the penetrant and therefore a higher diffusivity. Less soluble gases (e.g., N_2_ or O_2_) may show an almost constant diffusion coefficient with increasing pressure or even decreasing diffusivities as these gases do not induce a significant plasticization at the respective pressure level. Results obtained in this study for PIM-1 and the related MMM with CPSF-EtO show this expected behavior concerning pressure dependence of P and D_eff_. It must be noted that the diffusion coefficients determined from the time-lag (which is rather short for PIM-1) exhibit a larger error than the permeabilities and are more sensitive to pretreatment and history. Nevertheless, the presented data allow for a reliable assessment of the effect of the added nanofiller as well as plasticizing effects.

Figure 8a gives the permeabilities for CO_2_ for the different concentrations of CPSF-EtO in the MMMs. For all concentrations of CPSF-EtO a decrease of the permeability with increasing upstream pressure is observed, whereas the diffusion coefficients increase with increasing upstream pressure (Figure 8b). Such a dependence has been also reported earlier for neat PIM-1^36^ and is predicted by the dual-mode-sorption model. In this actual case, the decreasing gas solubility S following this model dominates the increasing diffusivity (due to the plasticizing effect of the rather high gas loading of CO_2_) resulting in a decrease of permeability P. Overall, with increasing the CPSF-EtO concentration, the permeability for CO_2_ increases reaching a maximum value for 7 wt % CPSF-EtO in the MMM. A further increase of the concentration of the filler content to 10 wt % leads to a decrease in the permeability and diffusion coefficient values. As observed for CO_2_, the CH_4_ permeability and diffusion coefficients increase with increasing filler content up to 7 wt % CPSF-EtO (Figure 9). The permeability data and diffusion coefficients for all gases and concentrations of the composites are given in Table S1. As for CO_2_ and CH_4_, permeability and diffusion coefficients of O_2_ for the MMMs loaded with 7 wt % filler are remarkably higher compared to neat PIM-1 (Figure 10). For N_2_, the permeability remains unchanged by adding the particles considering the error up to a concentration of 7 wt % of the filler. However, a loading of 10 wt % leads to a significant drop of permeability by 30% compared to unfilled PIM-1 (Figure 11a). A closer look at the relative change of diffusivity and permeability compared to the respective values of pure PIM-1 confirms the observed trend.

As discussed above, in the context of permeability of the investigated nanocomposites, one must expect two opposing effects. The incorporation of the CPSF-EtO filler fraction dispersed as isolated sheets may on one hand affect the packing of the polymer matrix and thus result in additional free volume facilitating the uptake and diffusion of penetrant molecules. On the other hand, the filler sheets may also block bottlenecks or apparently fill the microporous network of the PIM-1 matrix leading to the opposite effect. Here, one must keep in mind that the filler is already present when the solid morphology of the composite is formed from the casting solution. This means that the formation of the microporous network is affected by the filler rather than filled-up afterward as discussed above. Nevertheless, it is then reasonable to assume that the balance of the two phenomena depends on the overall filler concentration. Furthermore, the larger aggregates may induce additional free volume on a larger length scale but still in the nanometer range, as discussed in the morphology section (3.1). As these primary aggregates form even larger secondary aggregates, this may result in another concentration dependence of the matrix behavior with respect to gas transport properties.

**Figure 12:**
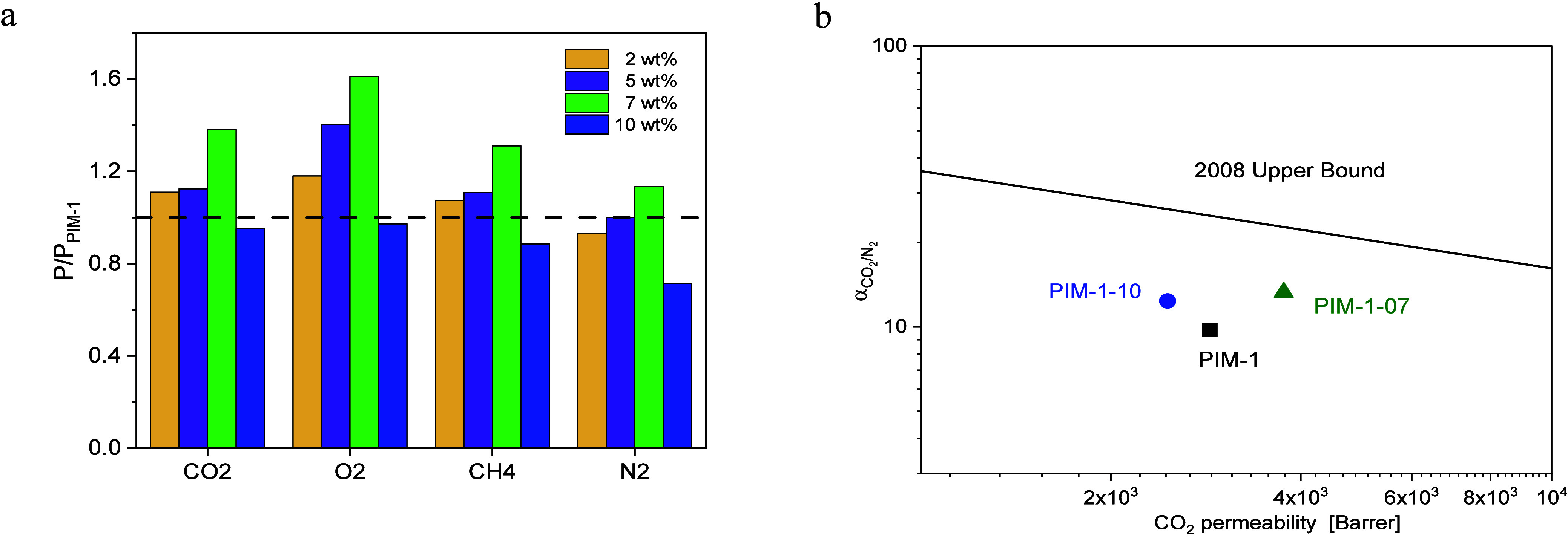
(a) Permeability of composites with different filler concentrations normalized by the permeability value of pure PIM-1 for the employed gases. (b) Selectivity for CO_2_/N_2_ versus the permeability of more permeable g CO_2_ for PIM-1 and the PIM-1–07 composite at 308 K and 1 bar. The solid line corresponds to Robeson upper bound 2008 adapted from ref (55). Here, the well-established 2008 upper bound is used for orientation and comparison as it is more common than the updated upper bound lines discussed, e.g., in ref (21). The selectivities for all considered samples and gas pairs are given in the Supporting Information in Table S1.

Overall, the investigated composites show a significant increase of permeability and diffusivity for all four measured gases with increasing filler content up to 7 wt %. For the higher filler concentration of 10 wt %, a distinct drop of these gas transport coefficients is obtained. The effect of the filler on the gas transport can be visualized by considering the observed changes in terms of permeability normalized in reference to the respective values of pure PIM-1 (Figure 12a). The changes in permeability follow qualitatively those of diffusivity, namely in dependence of gas as well as of filler content. The most significant increases of both, *D*_eff_ and *P*, are found for carbon dioxide and methane. On the other hand, nitrogen shows a distinct drop compared to the gas transport parameters of the other gases.

Based on the observations of the film morphology using T-SEM and X-ray scattering, this drop might be connected to the formation of secondary aggregates counteracting the effect of molecularly dispersed filler and of the primary aggregates dominating the behavior at the lower filler concentration levels.

The MMM containing 7 wt % CPSF-EtO deserves special attention, as it reveals pronounced increase in CO_2_ permeability by almost 50% compared to the unfilled polymer. The corresponding diffusivity coefficient *D* is approximately two times higher than that of neat PIM-1. Moreover, the *D*_eff_ values of all investigated gases for low filler concentrations are higher than that for neat PIM-1 probably due to an increase in the accessible free volume for gas molecules.

### Ideal Gas Selectivity of PIM-1/CPSF-EtO MMMs

The ideal gas selectivity for CO_2_ over N_2_ is estimated by using eq 2 and summarized in Table S1. As the filler content increases, the selectivity of CO_2_ over N_2_ increases. The values of ideal selectivity of CO_2_ and N_2_ are 11.3 and 13.7 for 7 and 10 wt % filler concentration, respectively. These values are 27% and 36% higher compared to that of neat PIM-1.

The significant increase in permeability (Figure 12a), especially for CO_2_, gives rise to improved performance regarding gas separation (Figure 12a). The increase in the permeability values for the composites compared to pure PIM-1 is ascribed to the additional free volume elements created by the filler. Also, the permselectivity is increased. This is illustrated in Figure 12b showing the ideal selectivity for CO_2_/N_2_ of PIM-1 with 7 and 10 wt % CPSF-EtO compared to pure PIM-1 and the 2008 upper bound in a Robeson plot. The increase in the selectivity values can be first discussed assuming that the created additional free volume elements are smaller than the initial micropores of PIM-1. Second, the X-ray data in the WAXS range indicate that also the microporous network is changed by the filler. This might also lead to an increase of the selectivity values.

It should be noted here that the PIM-1-based materials investigated in this study were not subjected to a post-treatment with alcohols, usually leading to higher permeability. This must be considered when comparing the obtained gas transport parameters to literature values.

Interestingly, the drop in permeability for 10 wt % CPSF-EtO is accompanied by a further increase in selectivity, thereby following the typical trade-off behavior.

### Physical Aging

**Figure 13:**
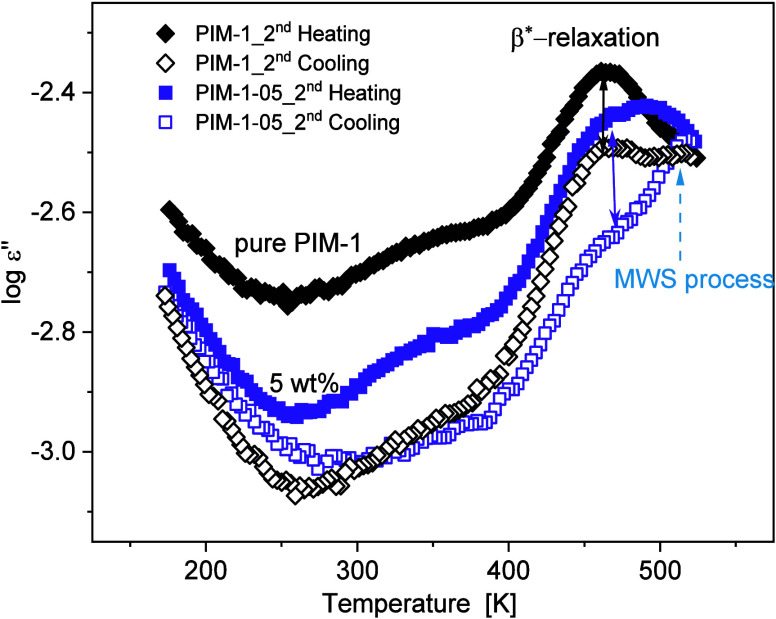
Dielectric loss spectra vs temperature for pure PIM-1 (black diamonds) and PIM-1–05 MMM (violet squares) at a fixed frequency (*f* = 1 kHz). Second heating (2H) and second cooling (2C) measurement cycles of BDS are shown with filled symbols and open symbols, respectively. The solid arrow indicates β*-relaxation peak position while the dashed arrow indicates MWS process.

Physical aging, which is an important issue for polymers of intrinsic microporosity, is relevant in two ways in the context of this study. At first, its effect is observed during the temperature ramp connected with the BDS measurements. Second, it is explicitly addressed regarding the long-term behavior of permeability and permselectivity. To investigate the influence of the temperature treatment during the measurement, the samples were measured by dielectric spectroscopy in two subsequent heating and cooling cycles in the temperature range of 173–523 K (with steps of Δ*T* = 3 K). In the first run, the samples were heated to 473 K and subsequently cooled to 173 K. This procedure was followed by the second heating run where the measurement was carried out up to 523 K and cooled down to 173 K in the subsequent second cooling run. To illustrate the dielectric behavior, the dielectric loss is plotted in Figure 13 versus temperature at the frequency of 1 kHz for the second heating and second cooling runs for pure PIM-1 and the MMM film with 5 wt % of the filler.

As mentioned earlier, the lower dielectric loss for the composite materials might be due to the lower molecular dipole moment of CPSF-EtO. For neat PIM-1, a significant reduction in the dielectric loss level is observed by comparing the dielectric loss spectra of the membrane for the second heating and second cooling cycle. At low temperatures, the pore network structure is static because there is no molecular mobility that can modify the microporous network structure. With increasing temperature, the β*-relaxation becomes activated at around 373 K. The static pore network becomes a dynamic one after the molecular mobility is introduced to the polymer which leads to restructuring of the pore network. It is obvious that this effect becomes more pronounced with increasing temperature. The changes induced by physical aging at this stage are directly related to the maximum temperature at which the material was exposed. Therefore, the drop in dielectric loss level for the second cooling run is attributed to the restructuring of the pore network due to the annealing conditions during the second heating run.^29^ Consequently, the comparison of the changes observed at this step may provide at least a first indication of the physical aging behavior. Overall, a smaller drop in dielectric loss is observed prior to and after exposure to high temperatures (up to 523 K) for the composite PIM-1–05 compared to the unfilled PIM-1.

A more detailed analysis reveals a significant decrease of the intensity of the peak attributed to β*-relaxation process for PIM-1–05 in the second cooling run, while for the neat PIM-1 a pronounced β*-relaxation peak remains also after annealing. Moreover, the peak of the MWS process for the second cooling run appears to be modified by the effect of an elevated temperature on the morphology of the filled membranes. The decrease in the intensity of the β*-relaxation for the PIM-1–05 membrane indicates that restructuring due to physical aging in the presence of CPSF-EtO fillers influences the fluctuations related to the intermolecular agglomerates of the aromatic moieties of the PIM-1 matrix.

Although drawing a straightforward conclusion is difficult due to the complexity of the dielectric relaxation spectra in the temperature range of the β*-and MWS processes, the incorporation of CPSF-EtO into the PIM-1 matrix leads to a more pronounced restructuring of the micropore network within the matrix than the effect of annealing.

**Figure 14:**
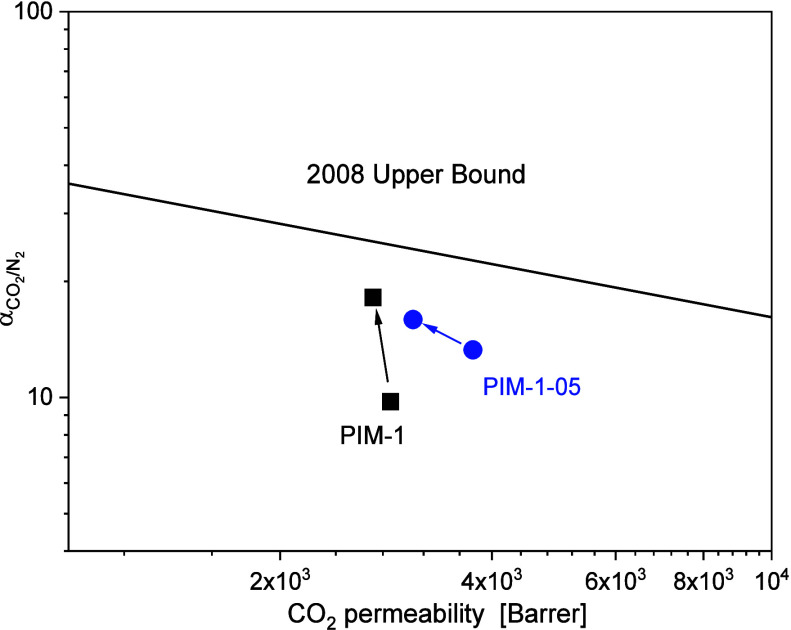
Selectivity for CO_2_/N_2_ vs the permeability of CO_2_ for PIM-1 and PIM-1–05 composite freshly prepared and after 270 days as indicated at 308 K and 1 bar. The solid line corresponds to Robeson upper bound 2008 adapted from ref (55).

In a second approach, the physical aging behavior was addressed in terms of the gas transport properties. To this aim, gas permeation properties for all gases under investigation were remeasured with a PIM-1–05 membrane that has been stored at room temperature for 270 days and compared with pure PIM-1 aged for the same period. The results are discussed in comparison with those obtained for the respective membrane in the freshly prepared state. In Figure 14 the CO_2_/N_2_ selectivity versus the CO_2_ permeability of PIM-1 and PIM-1–05 at 35 °C are shown in comparison to the 2008 upper bound at 1 bar. The performances of MMM and neat PIM-1 are below the 2008 upper bound and moved toward lower permeability and higher selectivity over time. After aging, the MMM with 5 wt % shows still a higher CO_2_ permeability in comparison to neat PIM-1 at the expense of a slight drop in CO_2_/N_2_ selectivity. As discussed earlier, one should consider that the properties of super glassy polymers and the aging behavior are affected strongly by different parameters such as membrane thickness and thermophysical history.

In this context, the special transport mechanism of PIMs, although still not completely understood, plays an important role. This peculiarity gives rise to the much better permselectivities of PIMs compared to other superglassy high-free volume polymers such as PTMSP. This manifests in a distinctly different dependence of diffusivity on the squared kinetic diameter of the penetrant gases. The latter represents the cross-sectional area of the diffusing molecule, determining the extent to which the surrounding matrix (i.e., the bottleneck between pre-existing voids) has to fluctuate in order to allow the penetrant to pass. The diffusion of small gases such as H_2_ or He is much less affected by this dynamic channel formation and therefore exhibits a lesser dependence on molecule size. Thus, the diffusion of these small penetrants has largely a character of transport through an interconnected micropore network, whereas larger penetrant gases experience much more size-selective transport according to the solution-diffusion mechanism. This behavior, described in detail by Fuoco et al.^72^ and being the reason for attractive separation performance, seems to be unique for PIMs and is not observed for example for PTMSP. Furthermore, this characteristic of PIMs has also an influence on the effect of aging on permeability and permselectivity. This can be visualized by comparing the aging-related shift of the data points in the Robeson diagram compared with the line of the upper bound as demonstrated by Bezzu et al.^73^

## Conclusions

This study investigated the molecular mobility and gas transport properties of PIM-1 mixed matrix membranes with CPSF-EtO as the nanofiller. It is worth noting that the CPSF-EtO in the bulk is not crystalline but amorphous and can be considered as a glass. The morphology of the prepared samples was investigated by two different methods, high-resolution scanning electron microscopy and X-ray scattering, providing different spatial resolutions. T-SEM images confirmed the filler embedded in PIM-1 matrices, presenting as largely dispersed COF structures or primary aggregates with sizes up to 100 nm. For higher nanofiller concentrations in the composites, larger secondary aggregates of the filler were observed. From these investigations, it is concluded that the nanofiller is almost homogeneously distributed in the samples. X-ray scattering analysis, employing a Monte Carlo fitting algorithm, quantified size distributions up to 62 nm, confirming the successful filler dispersion and incorporation on a nanoscopic level. Additionally, the WAXS region of the X-ray data indicated dispersed-level incorporation of CPSF-EtO, significantly altering the microporous network of the polymer matrix.

The molecular dynamics of PIM-1/CPSF-EtO composite membranes was studied by dielectric spectroscopy. As previously observed for the pure PIM-1 matrix, several dielectrically active processes were found for the MMM composite materials. In contrast to pure PIM-1, a broadened MWS interfacial polarization process was observed at elevated temperatures for all composites, which might indicate the presence of an additional mechanism for the blocking of the charge carriers which might originate from the interface between the aggregates and the matrix (MWS_filler_) as well as the pore walls of PIM-1, partially modified by the presence of the CPSF-EtO filler (MWS_pore_). The analysis showed that aggregates created further free volume elements at the interface of small CPSF-EtO aggregates to the PIM-1 matrix.

For pure PIM-1 relatively low values of the permeability were found, which is due to the sample preparation and the fact that no alcohol treatment was employed. Nevertheless, the observed changes reflect the effect of the incorporated filler on the gas transport properties. For the composites, the obtained data show that the separation performance of the MMMs has been enhanced in terms of permeability without significant loss in the selectivity. Incorporating CPSF-EtO led to higher permeability values for O_2_, CH_4_, and CO_2_ compared to pure PIM-1. The optimal filler loading at 7 wt % resulted in a roughly 50% increase in CO_2_ permeability and a 27% improvement in CO_2_/N_2_ selectivity. This performance enhancement was attributed to the free volume created by larger filler aggregates compensating for the diffusion pathways lost due to pore-blocking by domains of the filler molecules. In conclusion, it can be stated that nanoparticles of covalent aromatic organic polymer frameworks containing phosphinine and thienothiophen moieties can be successfully incorporated in a PIM-1 matrix. It was further shown that thereby physical aging can be reduced and the gas transport properties can be enhanced simultaneously. This approach by combining broadband dielectric spectroscopy with structural sensitive methods like X-ray scattering as well as gas transport experiments should be extended to further microporous polymers like PIM-EA-TB or high performance microporous polynorbornenes and further COF materials with higher BET surface area values. In addition, also the molecular mobility of the COF filler should be addressed, especially when at least partially noncrystalline.^50^

## Supporting Information Available

Synthesis of PIM-1; FTIR spectra for PIM-1, CPSF-EtO and PIM-1–05; TGA measurements, example for the time-lag measurements; histograms obtained from the fitting analysis of the X-ray data; example for the fitting of the dielectric data; table of the gas transport properties (PDF)
