# Supplementary material for: Molecular Mobility and Gas Transport Properties of Mixed Matrix Membranes Based on PIM-1 and a Phosphinine Containing Covalent Organic Framework
Source: Macromolecules. 2024 Feb 9;57(4):1829–45. doi: 10.1021/acs.macromol.3c02419 (PMC10902888; doi:10.1021/acs.macromol.3c02419)
Supplement: Supplementary file 1 — ma3c02419_si_001.pdf [file ma3c02419_si_001.pdf]

Supporting Information

Molecular Mobility and Gas Transport Properties of Mixed Matrix Membranes based on PIM-1 and a Phosphinine containing Covalent Organic Framework

Farnaz Emamverdi [a], Jieyang Huang [b], Negar Mosane Razavi [a], Michael J. Bojdys [b], Andrew B. Foster [c], Peter M. Budd [c], Martin Böhning [a], Andreas Schönhals [a,*]

[a] Bundesanstalt für Materialforschung und -prüfung (BAM), Unter den Eichen 87, 12205 Berlin, Germany

[b] Department of Chemistry, Humboldt University, Brook-Taylor Straße 2, 12489 Berlin, Germany

[c] School of Chemistry, University of Manchester, Manchester M13 9PL, UK

*Corresponding author: A. Schönhals, BAM Bundesanstalt für Materialforschung und -prüfung (Fachbereich 6.6), Unter den Eichen 87, 12205 Berlin, Germany; Tel. +49 30 / 8104-3384; Fax: +49 30 / 8104-73384; Email: Andreas.Schoenhals@bam.de

Synthesis

Synthesis The synthesis of the sample was carried out according to the procedure below, based on that reported by Du et al[1]:

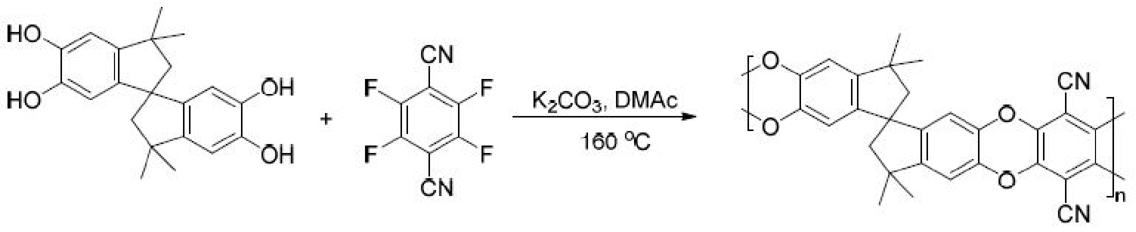

Scheem S1: Reaction scheme of PIM-1.

To a dry 500 ml three-necked round bottom flask equipped with a Dean-Stark trap, 5,5',6,6'-tetrahydroxy-3,3,3',3'-tetramethyl-1,1'-spirobisindane (TTSBI) (17.021 g, 0.05 mol), tetrafluoroterephthalonitrile (TFTPN) (10.005 g, 0.05 mol), anhydrous potassium carbonate (20.730 g, 0.15 mol), dimethylacetamide DMAc (100 mL), and toluene (50 ml) were added under an atmosphere of nitrogen gas. The monomers were allowed to dissolve before the reaction mixture was refluxed during rapid stirring at 200 rpm at 160 °C for 40 min. Heating was carried out using a IKA hot-plate together with a DrySyn aluminium heating block. After 40 min, the viscous solution was poured into methanol. To purify the polymer, the sample was dissolved in 500 ml of chloroform and re-precipitated in methanol while stirring. After washing with acetone, the product was stirred in 1,4-dioxane for 30 min to remove low molecular weight oligomers and cyclic products, before washing again with acetone. The sample was then refluxed overnight in deionized water, stirred in methanol for 20 min and then dried at 100 o C for two days. The final yield of PIM-1 obtained was 22.06 g (95.9 %).

SEC analysis: M_w = 106,200 g mol^{-1}, M_n = 59,100 g mol^{-1}, Dispersity = 1.80

Elemental analysis: C = 74.35 %, H = 4.41 % N = 6.08 %

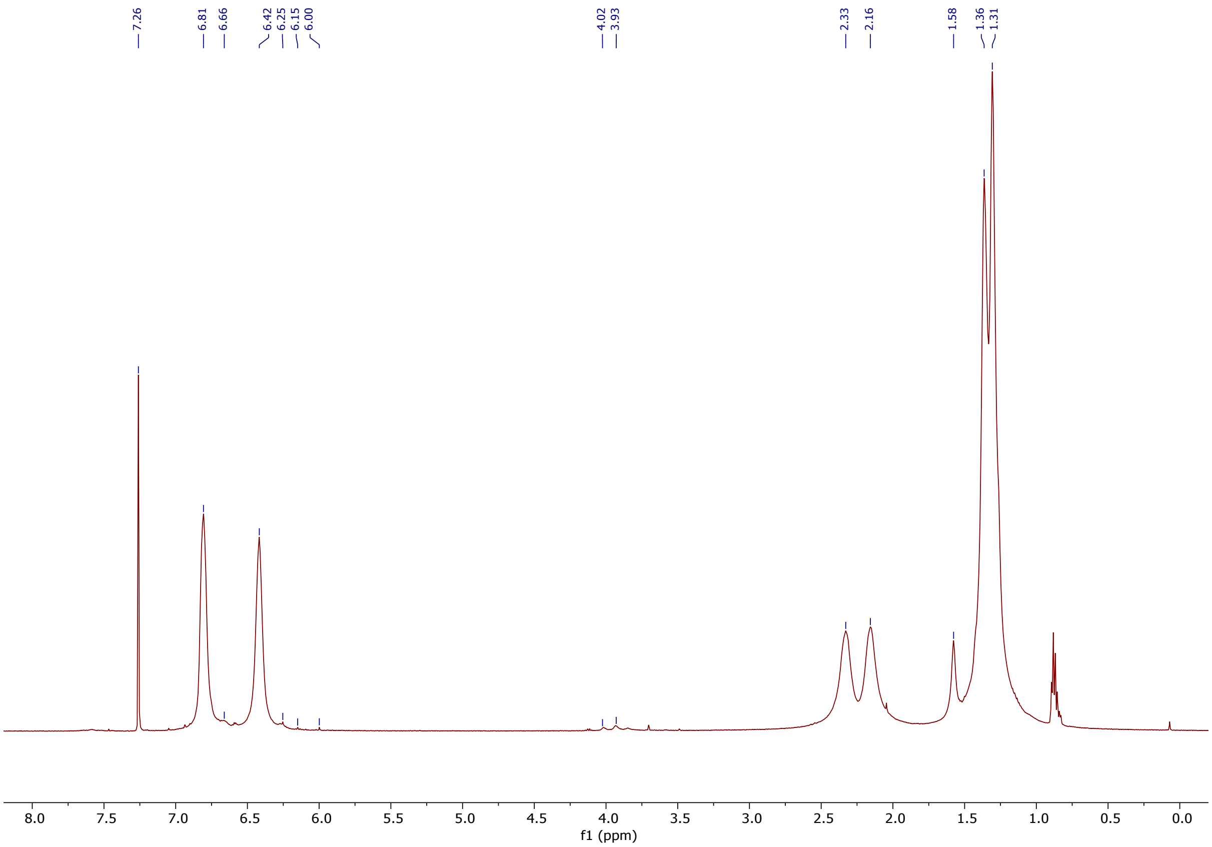

Figure S1. 1H NMR spectrum of PIM-1 sample

FTIR spectroscopy (ATR-IR)

ATR-FTIR spectroscopy was performed with a Nicolet 6700 equipped with SmartOrbit diamond module and DTGS detector. The spectra were recorded in a wavenumber range of 4500 to 450 cm^{-1}, averaging 32 scans with a resolution of 2 cm^{-1}. The obtained data were processed with ATR correction available with OMNIC 9 software (Thermo Fischer Scientific, Karlsruhe, Germany).

Results from FTIR

FTIR spectroscopy was performed to study the possible incorporation and interaction of the fillers in the PIM-1 matrix (Figure S2). H-bonds between the alkoxy group (O-CH_2CH_3) in EtO-CPSF and cyano ($-C\equiv N$) group can be formed. Such bonds are much weaker than the covalent bonds but stronger than the typical intermolecular van der Waals interactions[2]. The formation of H-bonds can shift the characteristic peaks to lower wavenumbers[3]. Nonetheless herein no significant changes were observed between the FTIR spectra of PIM/CPSF-EtO and neat PIM-1 membranes. This is possibly due to the presence of mostly proton accepting bonds in both filler and matrix.[4]

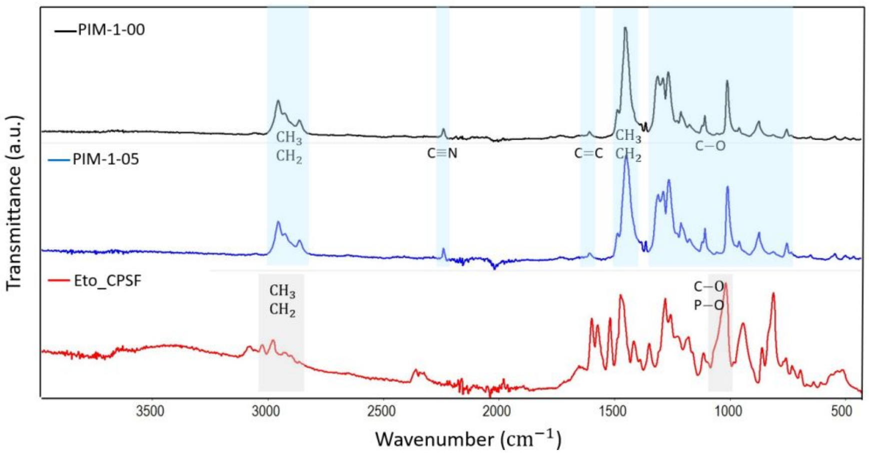

Figure S2. FTIR spectra of PIM-1, CPSF-EtO nanofiller and PIM-1/CPSF-EtO with 5wt% filler concentration.

Thermogravimetric analysis (TGA)

Thermogravimetric analysis was performed using a STA7000 Thermogravimetric Analyzers (Hitachi, Chidoya, Japan), employed with a horizontal dual balance. In the first TGA measurement samples of 3-5 mg were heated in alumina pans from room temperature to 1000°C (1273 K) at a heating rate of 10 K min^{-1}. Nitrogen was used as a purge gas up to approximately 600 °C (873 K) to avoid oxidation reactions. From 600 to 1000 °C (873 – 1023 K), oxygen was used as purge gas to oxidize the sample completely.

Results from TGA

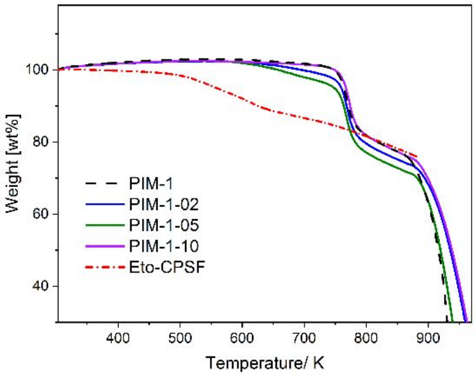

Figure S3. TGA curves of PIm-1, CPSF-EtO, and selected composites with 2, 5 and 10 wt% concentration of CPSF-EtO nanofiller.

According to the TGA results neat PIM-1 and the MMMs exhibit similar thermal stability in the studied temperature window (Figure S5). The small weight loss below 733 K corresponds to the removal of moisture and volatile organic compounds[5], whereas the significant weight loss at approximately 1000 K originates from the chemical degradation[6,7]. The TGA curve for pure CPSF-EtO reveals a lower thermal stability than PIM-1.

Time-lag curve

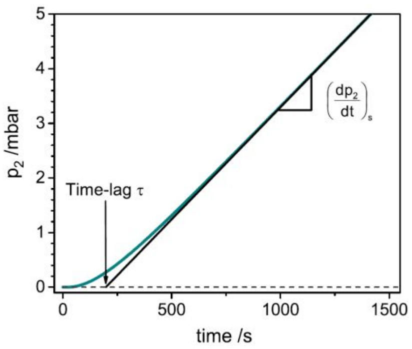

Figure S4. Exemplary time-lag curve for of Nitrogen gas: downstream pressure p_2 vs. time for pure PIM-1 at 35 °C and 3 bar.

X-ray Scattering

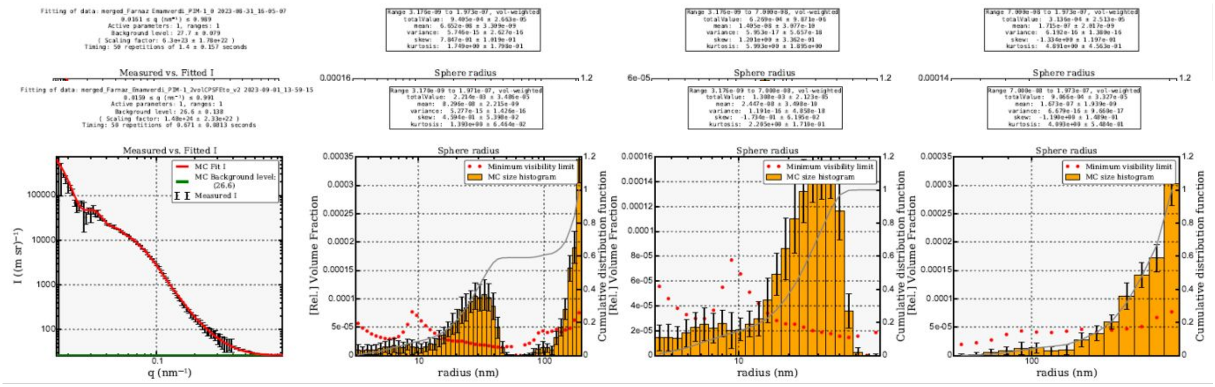

Figure S5. X-ray fitting results for PIM-1-02.

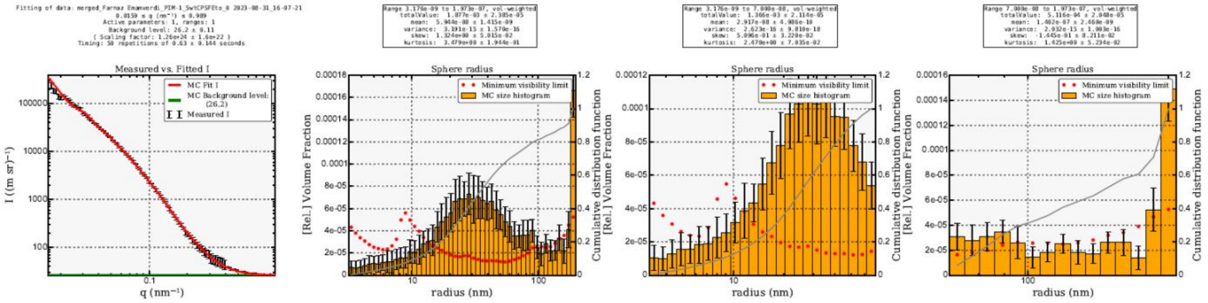

Figure S6. X-ray fitting results for PIM-1-05.

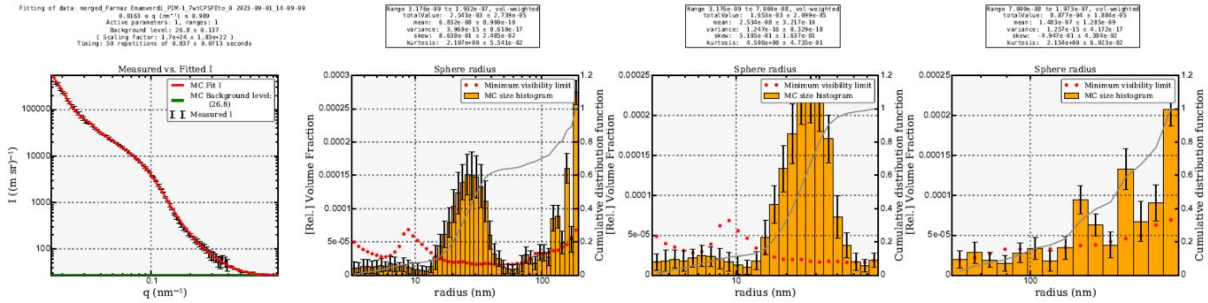

Figure S7. X-ray fitting results for PIM-1-07.

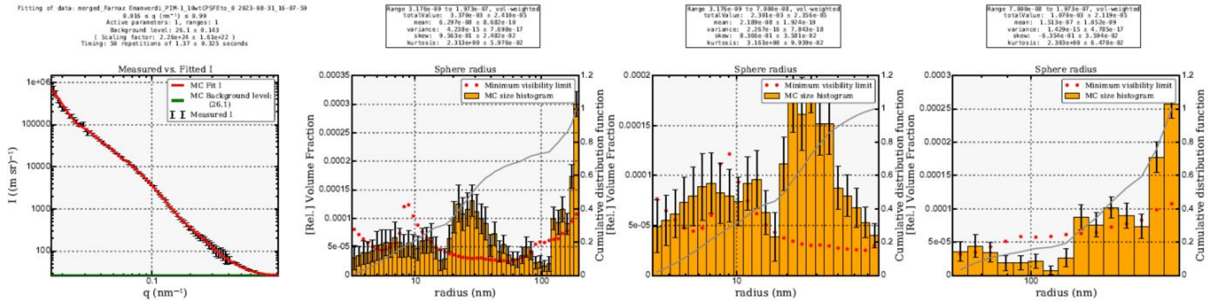

Figure S8**.** X-ray fitting results for PIM-1-10.

<u>Sample fit of the Havriliak-Negami (HN) function to the experimental data</u>

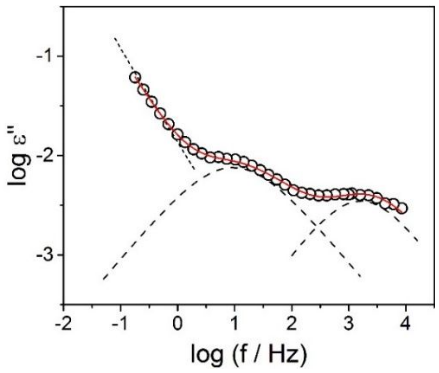

Figure S9. Example of HN-fits to the dielectric loss data. The solid red line represents a fit of two HN-functions and a conductivity contribution to the data for PIM-1 measured at T=473 K. The dashed lines depict the contributions of conductivity, β*- and β**-relaxation.

Permeability vs. kinetic diameter

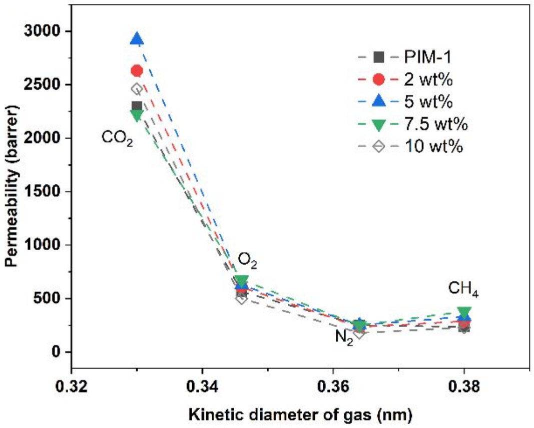

Figure S10. Permeability at 5 bar vs. kinetic diameter for all membranes.

Gas Transport properties

Table S1. Gas permeability (*P*), diffusion coefficient (*D*) obtained with the time-las setup for membranes at 35°C at 5bar.

Membrane	P (Barrer)	$D \times 10^{-7}$ $(cm^2 s^{-1})$	P (Barrer)	$D \times 10^{-7}$ $(cm^2 s^{-1})$	P (Barrer)	$D \times 10^{-7}$ $(cm^2 s^{-1})$	P (Barrer)	$D \times 10^{-7}$ $(cm^2 s^{-1})$	$\alpha_{CO2/N2}$
	CO_2		CH_4		O_2		N_2		

PIM-1	2340	8.84	236	2.53	566	19.55	252	9.85	9.08
PIM-1-02	2595	13.41	278.5	3.66	607.5	27.93	235	12.2	11.19
PIM-1-05	2630	13	331	4.25	627.5	25.78	252	11.6	11.58
PIM-1-07	3235	16.5	380	4.41	741.5	31.51	285.5	13.13	11.33
PIM-1-10	2225	11.31	229.5	2.82	501	21.3	180	6.91	12.36

References:

1 Du, N.; Robertson, G. P.; Song, J.; Pinnau, I.; Thomas, S.; Guiver, M. D. (2008). Polymers of intrinsic microporosity containing trifluoromethyl and phenylsulfone groups as materials for membrane gas separation. *Macromolecules*, 41(24), 9656-9662.

2 Kaplan, I. G.; Fraga, S.; Klobukowski, M.; Kryachko, E. S. (1986). Theory of molecular interactions. *Studies in Physical and Theoretical Chemistry*, Volume 42 of the Series Analytical Spectroscopy Library, Elesevier.

3 Pu, Y.; Yang, Z.; Wee, V.; Wu, Z.; Jiang, Z.; Zhao, D. (2022). Amino-functionalized NUS-8 nanosheets as fillers in PIM-1 mixed matrix membranes for CO2 separations. *Journal of Membrane Science*, *641*, 119912.

4 Palusiak, M.; Grabowski, S. J. (2002). Methoxy group as an acceptor of proton in hydrogen bonds. *Journal of Molecular Structure*, 642(1-3), 97-104.

5 Satilmis, B.; Budd, P. M. (2014). Base-catalysed hydrolysis of PIM-1: amide versus carboxylate formation. *RSC Advances*, *4*(94), 52189-52198.

6 Luque-Alled, J. M.; Ameen, A. W.; Alberto, M.; Tamaddondar, M.; Foster, A. B.; Budd, P. M.; Gorgojo, P. (2021). Gas separation performance of MMMs containing (PIM-1)-functionalized GO derivatives. *Journal of Membrane Science*, *623*, 118902.

7 Mohsenpour, S.; Ameen, A. W.; Leaper, S.; Skuse, C.; Almansour, F.; Budd, P. M.; Gorgojo, P. (2022). PIM-1 membranes containing POSS-graphene oxide for CO2 separation. *Separation and Purification Technology*, *298*, 121447.
